# Nano-Phytomedicine: Harnessing Plant-Derived Phytochemicals in Nanocarriers for Targeted Human Health Applications

**DOI:** 10.3390/molecules30153177

**Published:** 2025-07-29

**Authors:** Nargish Parvin, Mohammad Aslam, Sang Woo Joo, Tapas Kumar Mandal

**Affiliations:** 1 School of Mechanical Engineering, Yeungnam University, Gyeongsan 38541, Republic of Korea; nargish.parvin@gmail.com; 2 School of Chemical Engineering, Yeungnam University, Gyeongsan 38541, Republic of Korea; mohammadaslam13@yu.ac.kr; 3 Directorate of Research, Nehru Kendra, Mahatama Jyotiba Phule Rohilkhand University, Bareilly 243006, India

**Keywords:** nano-phytomedicine, phytochemical delivery systems, targeted drug delivery, stimuli-responsive nanocarriers, plant-derived nanotherapeutics

## Abstract

Phytochemicals from medicinal plants offer significant therapeutic benefits, yet their clinical utility is often limited by poor solubility, instability, and low bioavailability. Nanotechnology presents a transformative approach to overcome these challenges by encapsulating phytochemicals in nanocarriers that enhance stability, targeted delivery, and controlled release. This review highlights major classes of phytochemicals such as polyphenols, flavonoids, and alkaloids and explores various nanocarrier systems including liposomes, polymeric nanoparticles, and hybrid platforms. It also discusses their mechanisms of action, improved pharmacokinetics, and disease-specific targeting. Further, the review examines clinical advancements, regulatory considerations, and emerging innovations such as smart nanocarriers, AI-driven formulation, and sustainable manufacturing. Nano-phytomedicine offers a promising path toward safer, more effective, and personalized therapies, bridging traditional herbal knowledge with modern biomedical technology.

## 1. Introduction

### 1.1. Background of Phytochemicals in Traditional and Modern Medicine

Phytochemicals are biologically active secondary metabolites derived from plants, including polyphenols, alkaloids, terpenoids, and flavonoids. These compounds serve crucial roles in plant defense and have long been recognized for their therapeutic properties in traditional medicine systems such as Ayurveda, Traditional Chinese Medicine (TCM), and Indigenous medicinal practices worldwide [1]. Historically, plant-derived products like willow bark (source of salicin) and opium poppy (source of morphine) formed the basis for modern drug development [2]. The transition from ethnobotanical usage to evidence-based applications marked the beginning of phytochemistry as a discipline. In modern medicine, several plant-derived drugs have achieved clinical success, such as paclitaxel (from *Taxus brevifolia*) for cancer, artemisinin (from *Artemisia annua*) for malaria, and berberine (from *Berberis vulgaris*) for infections and metabolic syndromes [3]. With technological advancement in analytical chemistry and pharmacology, the molecular targets and mechanisms of these compounds have become more clearly defined. However, despite significant bioactivity, their clinical use remains restricted due to poor pharmacokinetics and variable efficacy in vivo. Nonetheless, rising interest in “natural” therapies and increased awareness of chronic disease management have renewed focus on plant-based compounds for their multi-target actions, low toxicity profiles, and immunomodulatory effects [4]. This renewed scientific interest sets the stage for improving delivery systems that can address the inherent limitations of phytochemicals and maximize their therapeutic potential in modern healthcare.

### 1.2. Limitations of Free Phytochemicals

Despite their promising biological activity, most free phytochemicals exhibit poor drug-like characteristics that hinder their clinical application. One of the most critical limitations is low aqueous solubility. Over 40% of plant-derived compounds have poor water solubility, which restricts their absorption in the gastrointestinal tract and reduces systemic circulation levels [5]. Additionally, once absorbed, many phytochemicals undergo rapid first-pass metabolism in the liver and intestines, leading to fast degradation and clearance from the body [6]. Another key issue is chemical instability. Many phytochemicals, such as curcumin and quercetin, are sensitive to light, pH, and enzymatic oxidation, resulting in the loss of bioactivity before reaching their target tissues [7]. Furthermore, poor membrane permeability and non-specific distribution limit their accumulation at disease sites while increasing the risk of side effects elsewhere. For instance, flavonoids with potent in vitro anticancer activity often fail in clinical trials due to these delivery challenges [8]. In essence, the therapeutic potential of phytochemicals is constrained by a triad of solubility, stability, and bioavailability issues. These challenges necessitate the development of advanced delivery systems such as nanocarriers that can protect the compounds, enhance absorption, and facilitate site-specific action to realize their full clinical value [9].

### 1.3. Emergence of Nanotechnology in Phytomedicine

**Figure 1:**
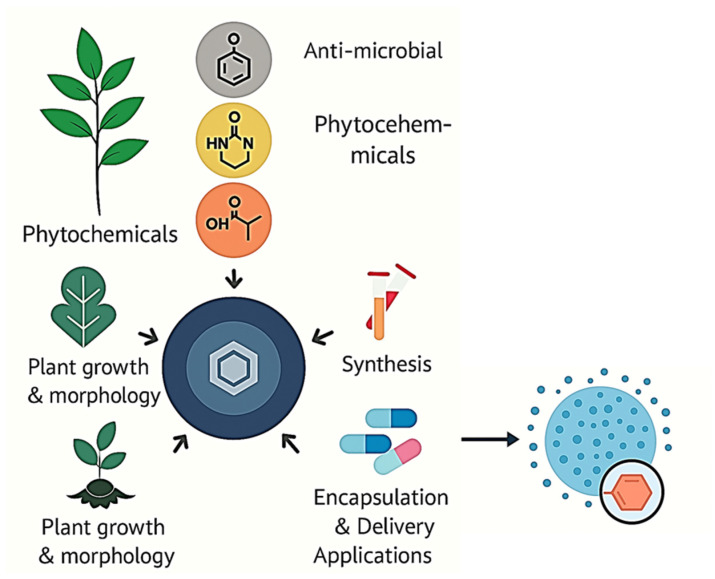
Emergence of nanotechnology in phytomedicine: from plant-derived bioactives to multifunctional nanoformulations.

Nanotechnology offers a transformative approach to improving the pharmacological performance of phytochemicals. By encapsulating these compounds in nanoscale carriers (typically 1–100 nm), researchers can significantly enhance their solubility, protect them from degradation, and extend their circulation time [10,11,12,13,14,15,16]. Nanocarriers like liposomes, solid lipid nanoparticles (SLNs), polymeric nanoparticles, dendrimers, and metallic nanostructures have shown promising results in stabilizing phytochemicals and enabling targeted delivery [17,18,19]. For example, encapsulating curcumin in polymeric nanoparticles has been shown to improve its bioavailability by over 2000%, while quercetin-loaded liposomes can deliver controlled release and site-specific targeting [20]. Furthermore, the surface of nanocarriers can be functionalized with ligands such as antibodies, peptides, or folic acid to ensure specific binding to target cells, improving therapeutic efficacy and reducing systemic toxicity [21]. Stimuli-responsive carriers can also trigger drug release in response to pH, temperature, or enzymatic activity at the disease site, enhancing precision medicine strategies. Additionally, green synthesis approaches using plant extracts in nanoparticle preparation provide eco-friendly and cost-effective routes to phytochemical stabilization [22,23]. As such, nanotechnology not only enhances the pharmacokinetics of plant-derived therapeutics but also opens the door to multifunctional treatment platforms capable of combining therapy with diagnostics (theranostics), paving the way for next-generation phytomedicines [24]. Figure 1 illustrates the transformative integration of nanotechnology into phytomedicine, emphasizing how plant-derived bioactive compounds are engineered into multifunctional nanoformulations for advanced therapeutic applications. Phytochemicals—such as polyphenols, alkaloids, and terpenoids—exhibit potent biological activities, but their clinical translation is often hindered by low solubility, instability, and poor bioavailability. Nanotechnology addresses these limitations through encapsulation strategies using various nanocarriers (e.g., liposomes, polymeric nanoparticles, nanoemulsions), which protect the phytochemicals, enhance cellular uptake, and provide controlled release. The schematic shows the central role of these carriers in enabling targeted delivery, reducing systemic toxicity, and facilitating precision medicine approaches. Furthermore, nanocarrier design can be tuned to respond to specific stimuli (pH, enzymes, redox conditions), offering spatiotemporal control over drug release. This convergence of botanical pharmacology and nanotechnology not only improves therapeutic efficacy but also supports the development of eco-friendly and biocompatible treatments with potential applications in oncology, neurodegeneration, and infectious diseases. The figure encapsulates this paradigm shift in drug delivery science, highlighting a new era of sustainable and personalized phytomedicine.

### 1.4. Objective and Scope of the Review

This review aims to provide a comprehensive overview of nano-phytomedicine, focusing on how nanotechnology can address the delivery challenges of phytochemicals and enable targeted therapy for various health conditions. The objectives are:

1. To categorize major nanocarrier systems including lipid-based, polymeric, and inorganic nanomaterials used in phytochemical encapsulation and delivery, with an emphasis on their physicochemical properties and compatibility with bioactives.
2. To elucidate molecular mechanisms of nano-phytochemical interactions with human cells, including uptake, intracellular trafficking, release kinetics, and downstream signaling modulation.
3. To discuss therapeutic applications across key areas such as oncology, neurodegeneration, cardiovascular disease, metabolic disorders, and infectious diseases, highlighting both preclinical and clinical outcomes.
4. To evaluate the pharmacokinetic and toxicological profiles of nanoformulated phytochemicals, considering parameters such as bioavailability, biodistribution, toxicity, and immunogenicity.
5. To assess the current translational landscape, including clinical trials, regulatory barriers, commercialized products, and future scalability for global healthcare implementation.
6. To explore future directions such as AI-guided formulation design, personalized nano-phytotherapy, smart delivery systems, and sustainable manufacturing.

By synthesizing recent advances and challenges, this review aims to offer insight into the integration of phytochemistry with nanotechnology, and how this convergence can revolutionize drug delivery and human health outcomes [25].

## 2. Overview of Plant-Derived Phytochemicals Relevant to Human Health

### 2.1. Major Classes: Polyphenols, Alkaloids, Terpenoids, Flavonoids, and Glycosides

**Figure 2:**
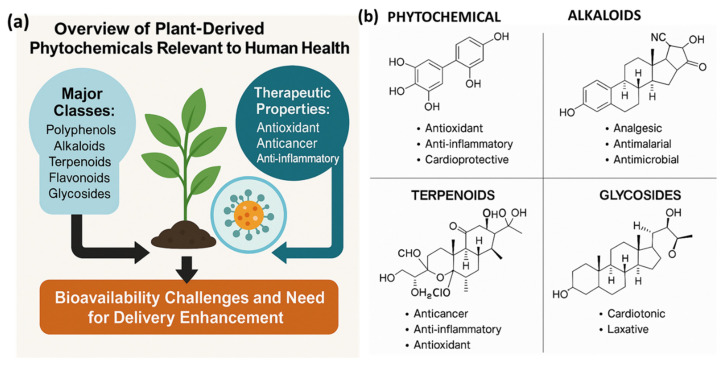
(**a**) Overview of plant-derived phytochemicals relevant to human health. (**b**), Chemical structures of major phytochemical classes polyphenols, alkaloids, terpenoids, flavonoids, and glycosides highlighting their therapeutic relevance. These scaffolds form the basis for nanoformulation strategies in modern phytomedicine.

Figure 2a provides an integrated schematic overview of the key classes of phytochemicals relevant to human health, including polyphenols, alkaloids, terpenoids, flavonoids, and glycosides, along with representative chemical structures and therapeutic roles. Figure 2b, representative chemical structures of major phytochemical classes including polyphenols (e.g., quercetin), alkaloids (e.g., berberine), terpenoids (e.g., paclitaxel), flavonoids (e.g., catechin), and glycosides (e.g., digoxin). These structural frameworks underpin diverse therapeutic activities, supporting their integration into nanoformulated delivery platforms for enhanced bioactivity and targeted treatment. These scaffolds form the basis for nanoformulation strategies in modern phytomedicine. Polyphenols are the largest family of plant secondary metabolites, defined by multiple phenolic rings and subdivided into phenolic acids, flavonoids, stilbenes, and lignans [26]. These compounds are abundant in dietary sources such as fruits, vegetables, tea, and wine. Flavonoids such as quercetin, catechins, and anthocyanins are especially prominent and contribute to cardiovascular protection, blood sugar regulation, and neuroprotection, as evidenced in numerous epidemiological and intervention studies [27]. Alkaloids, characterized by nitrogen-containing heterocycles, exhibit diverse pharmacological effects. Classic examples include morphine and codeine (opioid analgesics), quinine (antimalarial), and berberine (antimicrobial and metabolic regulator) [28]. Their mechanisms of action include receptor binding, enzyme modulation, and ion channel regulation at the molecular level. Terpenoids (isoprenoids), derived from five-carbon isoprene units, encompass subclasses such as monoterpenes (e.g., limonene), diterpenes (e.g., paclitaxel), and tetraterpenes (e.g., carotenoids). These exhibit potent anti-inflammatory, antioxidant, and anticancer properties with paclitaxel serving as a well-known chemotherapeutic agent [29]. Flavonoids, a major subclass of polyphenols, are multifunctional molecules capable of modulating oxidative stress, inflammation, mutagenesis, and carcinogenesis. They interact with key cellular pathways including NF-κB, MAPK, and PI3K/Akt, contributing to their observed protective effects in cardiovascular and cognitive health [30]. Glycosides, such as cardiac glycosides (e.g., digoxin) and anthraquinone glycosides (e.g., senna), play critical roles in modern therapeutics. Digoxin, isolated from *Digitalis*, enhances cardiac contractility via Na^+^/K^+^-ATPase inhibition, whereas anthraquinone glycosides stimulate intestinal motility, providing laxative benefits [31]. By depicting their representative molecular frameworks and associated health applications, Figure 2 contextualizes these chemical classes within modern phytomedicine. This visual integration renders a separate general subchapter unnecessary while enhancing reader comprehension.

### 2.2. Therapeutic Properties: Antioxidant, Anticancer, Anti-Inflammatory, Antimicrobial

Phytochemicals exhibit multiple overlapping therapeutic effects:

**Antioxidants**: Polyphenols neutralize free radicals and reactive oxygen species (ROS) in vitro, though their direct antioxidant activity in vivo is less certain due to poor bioavailability [32]. Still, their metabolites may indirectly upregulate endogenous antioxidant pathways via Nrf2 signaling.
**Anticancer activity**: Numerous phytochemicals modulate apoptosis, cell proliferation, angiogenesis, and metastasis. For example, catechin-3-gallate has been shown to enhance therapeutic efficacy through ROS induction and mitochondrial pathway activation in tumor cells [33]. Additionally, carrier-free self-assembled phytochemical nanoparticles (e.g., curcumin conjugates) demonstrate synergistic targeting and cytotoxicity in cancer models [34].
**Anti-inflammatory effects**: Myricetin, quercetin, and resveratrol inhibit pro-inflammatory cytokines such as IL-1β and TNF-α through downregulating signaling kinases and transcription factors (NF-κB, MAPK), showing promise in inflammatory and autoimmune disorders [35,36].
**Antimicrobial activity**: Polyphenol-rich plant extracts and alkaloids (like berberine) show broad-spectrum antimicrobial effects. For instance, berberine disrupts bacterial cell membrane integrity, while flavonoids inhibit viral enzymes and microbial adhesion [37].

These bioactivities highlight the multifaceted therapeutic potential of these phytochemicals—when adequately delivered and bioavailable.

### 2.3. Bioavailability Challenges and Need for Delivery Enhancement

Phytochemicals face significant barriers in clinical translation due to poor pharmacokinetic properties:

- **Poor solubility and stability**: Many are hydrophobic and insoluble in aqueous media, leading to limited absorption across the gastrointestinal tract [38]. Moreover, they are prone to degradation through oxidation, heat, light, or pH changes [39].
- **Rapid metabolism and clearance**: After absorption, phytochemicals are quickly subjected to phase I/II metabolism (e.g., glucuronidation, sulfation), yielding low plasma concentrations and short half-lives that necessitate frequent dosing [40].
- **Low membrane permeability**: Their molecular size, polarity, or lipophilicity often limits cellular uptake and penetration through biological barriers like the blood–brain barrier [41].
- **Variability in bioavailability**: Individual differences in gut microbiota, genetic polymorphisms in metabolic enzymes, and interactions with food can result in erratic absorption and therapeutic response [42].

**Figure 3:**
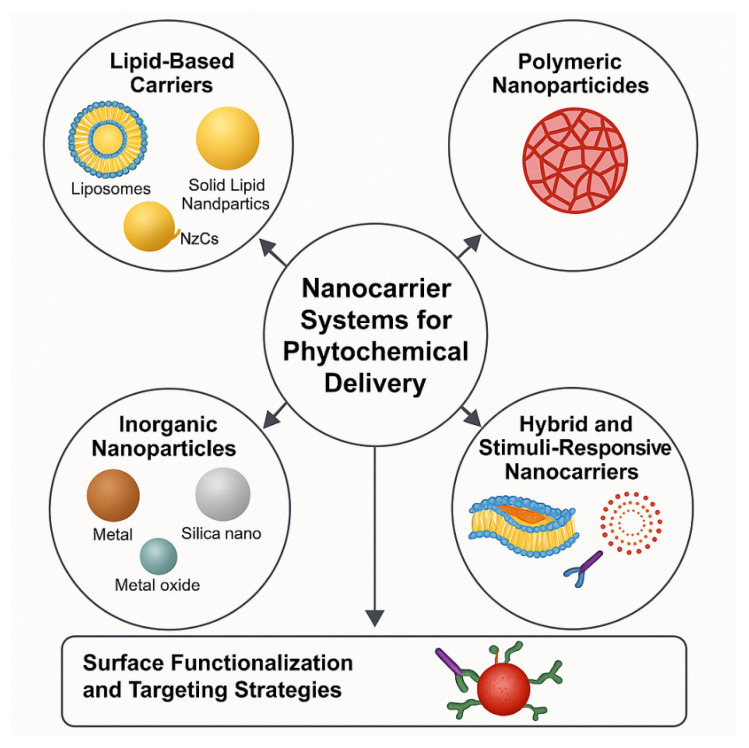
Comprehensive classification and functional overview of nanocarrier systems for phytochemical delivery.

As conventional dosage forms fail to resolve these issues, nanotechnology has emerged as a leading strategy. Nanocarriers including liposomes, polymeric nanoparticles, solid-lipid structures, chitosan-based carriers, nanoemulsions, and mesoporous silica provide tailored solutions: they enhance solubility, protect against degradation, facilitate controlled release, improve permeability, and enable target-specific delivery [43,44,45]. For example, chitosan nanoparticles administered orally increase mucoadhesion, protect from gastric enzymes, and facilitate transport across the intestinal epithelium [46,47]. In a comparative study, curcumin-loaded chitosan nanoparticles demonstrated a 7.4-fold increase in oral bioavailability compared to free curcumin. Mesoporous silica supports the stabilization of poorly soluble phytochemicals in amorphous form, boosting dissolution rates [48]. A study showed that mesoporous silica nanocarriers encapsulating resveratrol increased its aqueous solubility by over 10-fold and enhanced its antioxidant activity in vitro. Hybrid delivery systems combining multiple materials or nanocarrier types offer synergistic advantages. For instance, lipid-polymer hybrid nanoparticles encapsulating quercetin achieved enhanced cellular uptake and prolonged systemic circulation compared to either liposomes or polymeric carriers alone. Stimuli-responsive nanocarriers that release phytochemicals in response to environmental triggers (pH, temperature, redox state, or enzymes) are particularly promising. For example, pH-responsive nanoparticles carrying berberine showed selective release in acidic tumor microenvironments, improving cytotoxicity against cancer cells while sparing healthy tissue. In comparing delivery systems, studies have shown that nanoemulsions, while improving solubility, are less stable than solid lipid nanoparticles under physiological conditions. Conversely, polymeric nanoparticles provide controlled release but may show slower release kinetics compared to liposomal systems. These findings underscore the importance of tailoring nanocarriers to the specific physicochemical and therapeutic profile of each phytochemical. So the integrating nanocarrier technologies with in vitro and in vivo pharmacokinetic evaluations enables rational design for optimized therapeutic performance. Next Figure 3 provides a visual summary of these nanocarriers systems and their comparative performance metrics in enhancing phytochemical bioavailability. Table 1 presents a comparative overview of the major classes of plant-derived phytochemicals, highlighting representative compounds, their primary therapeutic effects, and key delivery challenges. Polyphenols such as curcumin and resveratrol are widely studied for their potent antioxidant and anticancer properties, yet their clinical efficacy is often limited by poor water solubility and rapid metabolism. Alkaloids like berberine and vincristine exhibit strong pharmacological activity, but their therapeutic use can be constrained by toxicity concerns at high doses. Terpenoids, including paclitaxel and carotenoids, offer antimicrobial and anticancer benefits but suffer from oxidative instability. Flavonoids, commonly found in fruits and vegetables, demonstrate neuroprotective and anti-inflammatory effects, though their bioavailability is hindered by rapid metabolism and poor membrane permeability. Lastly, glycosides such as digoxin are known for their cardiotonic effects but face challenges related to gastrointestinal degradation. Overall, the table underscores the need for innovative delivery strategies such as nanocarriers to overcome these limitations and harness the full therapeutic potential of phytochemicals.

## 3. Nanocarrier Systems for Phytochemical Delivery

### 3.1. Lipid-Based Carriers: Liposomes, Solid Lipid Nanoparticles (SLNs), and Nanostructured Lipid Carriers (NLCs)

Liposomes are spherical vesicles composed of phospholipid bilayers encapsulating an aqueous core. Typically ranging from 50 to several hundred nanometers, these biocompatible carriers are ideal for delivering both hydrophilic and hydrophobic phytochemicals [49]. Their structural flexibility allows oral, topical, and parenteral delivery, with the lipid bilayer protecting compounds like polyphenols and carotenoids from degradation. Liposomal encapsulation can substantially enhance bioavailability: for instance, liposomal curcumin formulations have shown significantly better plasma levels than free curcumin [50]. Active targeting is achievable through surface modification with ligands such as polyethylene glycol (PEG), antibodies, or specific peptides, enhancing selective uptake in target tissues [51].

Solid Lipid Nanoparticles (SLNs) consist of solid lipids stabilized by surfactants, offering stability at room and body temperatures. SLNs combine the benefits of lipid emulsions and polymeric carriers, such as controlled release, protection against chemical degradation, and scalable manufacturing, while avoiding organic solvents [52]. They can incorporate both lipophilic and hydrophilic phytochemicals, resisting filtration by the mononuclear phagocyte system when optimized at 120–200 nm [53]. SLNs have been shown to improve oral bioavailability of compounds like ferrous sulfate and lipophilic phytochemicals via enhanced gastrointestinal stability and absorption [54].

Figure 3 provides a comprehensive illustration of the diverse nanocarrier systems employed for phytochemical delivery, categorized into lipid-based, polymeric, inorganic, hybrid, and stimuli-responsive platforms. Lipid-based systems such as liposomes, solid lipid nanoparticles (SLNs), and nanostructured lipid carriers (NLCs) are shown to enhance solubility, protect against degradation, and facilitate passive targeting via the enhanced permeability and retention (EPR) effect. Polymeric nanoparticles, crafted from both natural (e.g., chitosan, alginate) and synthetic (e.g., PLGA, PEG) polymers, offer tunable release kinetics and surface modifications for site-specific delivery. Inorganic carriers including metal and metal oxide nanoparticles (like gold, silver, and ZnO) and mesoporous silica are highlighted for their intrinsic bioactivity, stability, and potential for imaging-guided therapy. Hybrid systems integrate lipid, polymer, or inorganic components to optimize payload encapsulation and dual functionality. Stimuli-responsive nanocarriers, which release their phytochemical load upon exposure to triggers such as pH, redox potential, or enzymes, provide controlled and precision-targeted therapeutic action, marking a pivotal innovation in nano-phytomedicine.

Nanostructured Lipid Carriers (NLCs) are advanced lipid-based systems containing mixed solid and liquid lipids to increase payload capacity and reduce crystallinity. This design enhances drug loading flexibility and release control [55]. NLCs demonstrate improved encapsulation efficiency and sustained release profiles over SLNs, making them well-suited for phytochemicals prone to rapid degradation. Preclinical studies have shown that NLCs loaded with botanical extracts achieve higher stability and biodistribution compared to free compounds, underscoring their potential for targeted delivery [56]. Lipid-based carriers thus offer protective encapsulation, improved dissolution, controlled release, and opportunities for functionalization. These capabilities position them as leading platforms to address solubility, stability, and targeted delivery challenges intrinsic to phytochemical therapies [57,58,59].

### 3.2. Polymeric Nanoparticles: Natural and Synthetic Polymers

Polymeric Nanoparticles (PNPs) are colloidal systems prepared from biodegradable polymers, offering robust strategies for encapsulating, protecting, and delivering phytochemicals [60]. These systems can be engineered as nanospheres, with bioactives dispersed within the polymer matrix, or nanocapsules, with a distinct core–shell architecture encapsulating the compound [61]. Additional morphological variants include micelles, dendrimers, and polyplexes, each customizable for specific payloads and release mechanisms [62]. Natural polymers such as polysaccharides (e.g., chitosan, alginate) and proteins (e.g., albumin, gelatin) offer high biocompatibility, biodegradability, and lower immune reactivity. Chitosan, in particular, enhances mucoadhesion and opens tight junctions in epithelial tissues, improving oral delivery of nutrients and phytochemicals [63,64]. Albumin and gelatin are widely used for their high binding capacity and ease of functionalization. For example, albumin nanoparticles loaded with berberine or ginsenosides have demonstrated increased circulation time, tumor targeting, and anticancer activity compared to free compounds [65,66]. Synthetic polymers like PLGA, PLA, PCL, and PEGylated derivatives are prized for their tunable degradation rates, mechanical properties, and reproducibility [67,68]. PLGA nanoparticles have been employed for curcumin, EGCG, and lycopene delivery, employing techniques such as emulsion diffusion and nanoprecipitation. In a recent study, lycopene-loaded PLGA NPs achieved encapsulation efficiencies of about 97%, with controlled release over 12 days [69]. PLGA nanoparticles also enable spray-dry formulations and scale-up processes essential for industrial application [70,71]. Hybrid systems, such as polymer–lipid hybrids (PLHNPs), combine the stability and payload capacity of polymeric cores with the encapsulation efficiency and biocompatibility of lipid shells [72]. PLHNPs have shown improved encapsulation rates, protection against enzymatic breakdown, and enhanced in vivo stability for phytochemicals [73]. Overall, polymeric nanocarriers provide highly tunable platforms capable of high drug loading, controlled and stimuli-responsive release, surface functionality for targeting, and favorable pharmacokinetic profiles. Such versatility makes them core components in the toolbox of nano-phytomedicine [74,75].

### 3.3. Inorganic Nanoparticles: Metal, Metal Oxide, and Silica Nanoparticles

Inorganic nanoparticles offer robust platforms for phytochemical delivery, characterized by unique physicochemical properties such as controlled size, surface charge, and imaging compatibility. Metal nanoparticles like gold (AuNPs) and silver (AgNPs) can be synthesized via green methods using phytochemicals both as reducing agents and capping ligands (e.g., flavonoids or polyphenols), allowing dual functionality: structural stabilization and therapeutic activity [76,77]. AuNPs functionalized with catechin or quercetin enhance targeted delivery and provide photothermal capabilities for cancer therapy [78]. AgNPs exhibit strong antimicrobial activity due to reactive oxygen species (ROS) generation, and when combined with phytoconstituents, they achieve a synergistic effect against resistant pathogens [79,80]. Metal oxide nanoparticles such as zinc oxide (ZnO) and iron oxide (Fe_3_O_4_) offer specialized functionalities. ZnO-NPs have intrinsic antimicrobial properties and can be loaded with curcumin or other bioactives to enhance wound healing, leveraging the combined effect of controlled drug release and inherent surface reactivity [81]. Fe_3_O_4_-based particles serve as drug carriers with magnetic targeting abilities, enabling external guidance and enhanced delivery efficiency for phytochemicals like resveratrol in tumor tissues [82]. Silica nanoparticles, particularly mesoporous silica nanoparticles (MSNs), are favored for their high surface area, tunable pore sizes, and ease of functionalization. MSNs can protect encapsulated phytochemicals from harsh gastrointestinal conditions, promoting controlled release profiles for hydrophobic compounds such as green tea catechins [83]. Functional moieties like pH-sensitive groups can be grafted onto the surface to achieve stimuli-responsive delivery ideal for tumor microenvironments or inflamed tissues [84]. Inorganic carriers thus enable multifunctional delivery and theranostic capabilities when combined with imaging agents or responsive triggers [85,86].

Table 2 illustrates the expanding versatility of nanocarrier platforms in phytochemical delivery, highlighting a strategic shift in formulation science from passive encapsulation to functionally adaptive systems. Beyond their individual characteristics, what emerges is a trend toward modularity and hybridization designing systems that merge structural integrity with responsiveness to biological cues. This adaptability allows for greater control over release kinetics and biodistribution, critical for maximizing therapeutic efficacy while minimizing systemic exposure. Notably, the incorporation of targeting ligands or stimuli-responsive elements signifies an evolution from conventional carriers to “smart” delivery vehicles. These advancements align with personalized medicine objectives, enabling site-specific delivery in heterogenous pathological conditions like tumors or inflamed tissues. Moreover, as nanocarrier designs increasingly integrate diagnostic and therapeutic functions, a new class of theranostic phytomedicines is emerging, capable of simultaneous treatment and monitoring. These trends underscore the importance of interdisciplinary innovation in driving the next generation of natural product-based therapeutics.

### 3.4. Hybrid and Stimuli-Responsive Nanocarriers

Hybrid nanocarriers combine organic and inorganic materials to achieve synergistic effects: improved payload stability, controlled release, and multifunctionality. One outstanding example is lipid–polymer hybrids, featuring a polymeric core for structural integrity and a lipid shell to improve biocompatibility and cellular uptake [87]. For instance, curcumin–PLGA core with a lecithin–PEG shell exhibits greater circulation time, tumor selectivity, and controlled release compared to single-material systems [92]. Gold-coated liposomes or polymeric nanocapsules can integrate photothermal functionality with drug delivery: upon laser irradiation at specific wavelengths, these constructs trigger localized heating that releases phytochemicals in target tissues a strategy beneficial in combined cancer therapy [93,94]. Similarly, magnetic-lipid hybrids incorporate iron oxide cores with lipidic envelopes to allow magnetic guidance and enhanced permeability in tumor regions [95]. Stimuli-responsive nanocarriers release their payload in response to internal (pH, enzymes, redox state) or external triggers (light, magnetic fields, ultrasound), thereby improving precision and therapeutic index. For example, pH-sensitive polymeric micelles release curcumin preferentially within the acidic tumor microenvironment, and enzyme-responsive systems such as matrix metalloproteinase (MMP)-cleavable linkers enable drug liberation only in inflamed tissues [88,89]. Redox-responsive carriers exploit high intracellular glutathione levels to selectively release encapsulated alkaloids like berberine within cancer cells [96]. These hybrid and stimuli-responsive systems are at the forefront of targeted nano-phytomedicine, with the potential to significantly elevate therapeutic outcomes.

### 3.5. Surface Functionalization and Targeting Strategies

Targeted nanocarrier delivery hinges on surface engineering. Passive targeting, driven by the enhanced permeability and retention (EPR) effect in tumor vasculature, is enhanced by PEGylation, which prolongs systemic circulation and reduces opsonization [90]. Active targeting uses ligands like folic acid, peptides (e.g., RGD), antibodies, or aptamers to direct particles towards cells expressing specific receptors. Folic acid-conjugated nano-structured lipid carriers loaded with resveratrol preferentially accumulate in folate receptor-overexpressing ovarian cancer cells, increasing uptake by over twofold and enhancing cytotoxicity [91]. Mannose or lectin surface decoration targets macrophages for anti-inflammatory phytochemicals like curcumin or quercetin in diseases such as arthritis and inflammatory bowel disease [97]. Antibody-conjugated carriers such as HER2-targeted polymeric nanoparticles loaded with paclitaxel precursor phytochemicals have shown improved selectivity and cytotoxicity in breast cancer models [98]. Stealth coatings using zwitterionic polymers or cell-membrane camouflaging (e.g., leukocyte or platelet membranes) further reduce immune recognition and improve circulation persistence [99]. Multifunctional or dual-targeting constructs allow for sequential targeting of vasculature and cancer cells by co-presenting antibodies and peptides, thereby enhancing tumor penetration [100]. Through judicious surface functionalization, nano-phytomedicines achieve a high degree of specificity and cellular control compared to conventional phytochemical administration methods.

## 4. Molecular Mechanisms of Action of Nano-Phytomedicine

### 4.1. Cellular Uptake and Intracellular Trafficking of Nanoformulations

Cellular uptake of nano-phytomedicines is governed by both the intrinsic properties of the nanocarriers such as size, surface charge, and ligand decoration and the specific endocytic pathways prevalent in target cells. Nanoparticles sized between ~50–200 nm are preferentially internalized via clathrin- and caveolin-mediated endocytosis, as well as macropinocytosis, allowing efficient intracellular delivery of phytochemicals [101]. Charge also plays a key role: positively charged nanocarriers interact more readily with negatively charged phospholipids, facilitating adsorption-mediated endocytosis. However, excessive surface cationicity must be balanced to avoid cytotoxicity and rapid clearance [102]. Surface functionalization with targeting moieties such as antibodies, peptides, aptamers, or small molecules (e.g., folic acid) directs nanoparticles to specific cell types. Ligand-receptor binding triggers receptor-mediated endocytosis, conferring selectivity and minimizing off-target distribution. Once internalized, trafficking routes diverge depending on nanosystem design. Lipid nanocarriers and polymeric vesicles may fuse with early endosomes, traverse the endosomal–lysosomal pathway, and eventually release their cargo via pH-responsive mechanisms. In contrast, certain inorganic or polymeric systems incorporate endosomal escape groups—such as proton-sponge domains or fusogenic peptides to release phytochemicals directly into the cytosol, avoiding degradation [103].

Intracellular trafficking also intersects with trafficking to subcellular organelles. Targeting mitochondria, endoplasmic reticulum, or even the nucleus often requires nanosystems equipped with organelle-specific peptides (e.g., TAT peptide, mitochondrial signal sequences). Mitochondria-targeted nano-phytomedicines can amplify ROS-mediated apoptosis specifically in cancer cells, while ER-targeted delivery can disrupt calcium homeostasis and protein synthesis. Moreover, nanoparticle core degradation kinetics determine whether phytochemicals are released early within lysosomes or later during cytosolic diffusion. Nanocarrier design also accounts for efflux mechanisms particularly P-glycoprotein pumps that expel small-molecule phytochemicals. Encapsulation protects the payload during uptake and retention, inhibiting efflux and prolonging intracellular exposure. For example, curcumin-loaded polymeric micelles show sustained cytosolic presence despite P-gp expression [104]. Furthermore, nanoparticle-mediated phytochemical delivery often modulates nutrient-sensing pathways (like mTOR and AMPK) post-uptake, enhancing uptake rates in proliferative tissues such as tumors. Overall, successful nano-phytomedicine design hinges on understanding and controlling cellular uptake and trafficking dynamics. Pairing size and surface chemistry with targeting ligands and endosomal escape strategies optimizes intracellular delivery, subcellular targeting, and payload release—foundations critical to maximizing therapeutic potential across diverse phytochemicals [105].

### 4.2. Modulation of Redox Balance and Antioxidant Pathways

Redox homeostasis plays a central role in disease progression, with an imbalance between reactive oxygen species (ROS) and cellular antioxidant defenses contributing to conditions ranging from cancer and neurodegeneration to chronic inflammation. Nano-phytomedicines leverage the inherent antioxidant properties of phytochemicals such as polyphenols and flavonoids while overcoming limitations in solubility and stability [106]. Encapsulation enhances intracellular accumulation of antioxidants, enabling robust modulation of redox-sensitive signaling pathways. A canonical pathway modulated by antioxidant phytochemicals is the Nrf2-Keap1 axis. Under oxidative stress, stabilized Nrf2 translocates to the nucleus and induces expression of phase II enzymes like glutathione-S-transferases and NAD(P)H:quinone oxidoreductase 1. Nanoformulations of resveratrol, quercetin, and sulforaphane have demonstrated enhanced Nrf2 activation compared to free forms, leading to improved cellular resilience and reduced oxidative damage in vitro and in vivo [107]. Mechanistically, sustained intracellular release from nanocarriers ensures prolonged antioxidant stimulation. In addition, nanocarrier encapsulation enables phytochemicals to neutralize ROS directly within subcellular compartments. For example, mitochondria-targeted nanocurcumin interrupts ROS generation in situ, preserving membrane integrity and preventing cytochrome-c release. Similarly, cerium oxide or iron oxide-based hybrid carriers can be functionalized or conjugated with phytochemicals to create synergistic antioxidant platforms. In inflammatory pathologies, redox modulation by these nanoconjugates intersects with pro-inflammatory signaling. Nano-phytomedicines that scavenge ROS also suppress NF-κB and MAPK pathways central regulators of cytokine production. For instance, epigallocatechin gallate (EGCG)-loaded polymeric nanoparticles reduce TNF-α and IL-6 release under oxidative stress by both direct radical scavenging and indirect gene modulation [108]. Despite the strength of these effects, accurate modulation demands balance. Over-suppression of ROS can impair immune functions, as controlled ROS levels are required for phagocytosis and pathogen defense. Advanced nanocarriers with redox-feedback sensitivity—releasing payloads only under high ROS—help mitigate this risk. For example, disulfide-containing polymeric carriers release phytochemicals specifically in oxidized environments, sparing normal cells from unnecessary antioxidant exposure [109]. So the nano-phytomedicine systems offer enhanced redox control by delivering antioxidants in targeted and controlled fashions. By focusing antioxidant activity where and when needed guided by biological cues these systems promise therapeutic benefits with reduced side effects, making redox modulation a cornerstone of phytochemical nanotherapy [110].

### 4.3. Regulation of Apoptosis, Cell Proliferation, and Inflammation

Nano-phytomedicines exert their therapeutic influence by orchestrating key cellular signaling pathways involved in apoptosis, proliferation, and inflammation. Unlike monotherapeutic small molecules, phytochemicals often achieve multitarget modulation, and nanoformulations enhance these effects by improving concentration and site-specificity at cellular targets [111]. A hallmark target in oncology is the intrinsic mitochondrial apoptosis pathway. Encapsulated curcumin, resveratrol, and berberine delivered via nanocarriers can accumulate in mitochondria, where they trigger cytochrome c release, upregulate pro-apoptotic proteins (Bax, Bak), and inhibit anti-apoptotic Bcl-2 family proteins. Nanodelivery increases mitochondrial targeting, leading to robust caspase-9 and -3 activation and apoptosis in cancer cells [112]. This selective activation in tumor cells helps reduce systemic toxicity. In parallel, cell proliferation pathways—particularly PI3K/Akt and MAPK/ERK are frequently dysregulated in disease. Nano-phytomedicines loaded with quercetin or apigenin have demonstrated potent inhibition of Akt phosphorylation and downstream mTOR signaling, reducing cell viability and inducing cell cycle arrest at G0/G1 in carcinoma models. Nanocarrier-enhanced retention ensures sustained exposure—critical for pathway inhibition [113]. Inflammation is another crucial target. Phytochemicals like curcumin and luteolin suppress NF-κB signaling, which regulates pro-inflammatory gene expression. Nano-phytomedicine systems amplify this effect by facilitating cytosolic uptake and nuclear translocation of inhibitory phytochemicals. Liposomal curcumin, for instance, reduces p65 phosphorylation more effectively than free curcumin, leading to downregulation of COX-2, iNOS, TNF-α, and IL-1β [114]. Furthermore, nano-phytomedicines can exert epigenetic effects—modulating histone deacetylases (HDACs), miRNAs, and DNA methylation—to sustain therapeutic responses. Resveratrol-loaded polymeric nanoparticles have been shown to upregulate miR-34a in tumor cells, promoting tumor suppressor gene expression and inhibiting proliferation [115]. Inflammasome activation represents another emerging mechanism. Nanocarriers delivering berberine or curcumin inhibit NLRP3 inflammasome assembly, reducing IL-1β secretion—a valuable action in autoimmune and metabolic diseases [116]. However, achieving specificity in apoptosis and proliferation regulation requires fine-tuning nanoparticle release profiles and cell selectivity. Overactivation of apoptosis in normal tissues can cause toxicity, emphasizing the importance of tumor-targeted nanoparticles and stimuli-responsive release systems. Overall, nano-phytomedicines modulate key cellular regulatory nodes apoptosis, proliferation, and inflammation—through optimized delivery of bioactive compounds. By controlling when, where, and how these pathways are targeted, nanocarrier systems amplify therapeutic efficacy while minimizing harm to healthy tissues [117].

### 4.4. Interaction with Cellular Signaling

Nano-phytomedicine systems exhibit profound capacity to modulate key intracellular signaling pathways that govern critical cellular processes such as growth, survival, stress response, and inflammation. Encapsulation of phytochemicals enhances their bioavailability and enables targeted delivery, ensuring pathway-specific engagement at therapeutically relevant concentrations [118]. The PI3K/Akt signaling axis plays a central role in cell survival, metabolism, and proliferation, and is often hyperactive in cancer and metabolic disorders. Phytochemicals such as curcumin, resveratrol, and quercetin are known inhibitors of this pathway. Nanoformulations further amplify these effects: for instance, quercetin-loaded polymeric nanoparticles achieve sustained Akt inhibition in breast cancer cells, reducing phosphorylated Akt levels significantly more than free quercetin [119]. Moreover, targeted nanocarriers allow co-delivery of PI3K/Akt inhibitors and phytochemicals, enabling synergistic suppression of proliferative signaling. The MAPK cascade including ERK, JNK, and p38 is triggered in response to mitogenic or stress signals. Phytochemicals like apigenin, kaempferol, and luteolin can selectively modulate MAPK sub-pathways. When delivered via nanoparticles, these compounds achieve higher intracellular concentrations, enhancing suppression of aberrant MAPK signaling. For example, liposomal apigenin reduces ERK phosphorylation in melanoma models, leading to growth arrest and apoptosis [120]. Redox-active phytochemicals in stimuli-responsive carriers also activate stress-activated kinases (e.g., p38, JNK), inducing apoptotic pathways in tumor cells. NF-κB is a master regulator of inflammation and cell survival. Persistent activation is implicated in chronic inflammatory diseases and cancer. Encapsulated curcumin, resveratrol, and EGCG delivered via liposomes or polymeric micelles exhibit enhanced NF-κB inhibition compared to free compounds. Nanocarrier-based delivery results in significant reduction in IκBα degradation and nuclear translocation of NF-κB subunits under inflammatory stimuli [121]. In macrophage and endothelial cell models, nano-phytomedicines reduce downstream cytokine secretion (TNF-α, IL-6), demonstrating functional pathway modulation [122]. By employing nanocarriers, researchers can tailor phytochemical dosing to durably modulate signaling without systemic toxicity. Temporal control via sustained release helps maintain signaling inhibition without triggering compensatory feedback loops. Surface functionalization with targeting ligands ensures preferential delivery to cells overexpressing receptors associated with specific signaling pathways. However, signaling pathway modulation is highly context-dependent. Nanocarrier design must account for tissue-specific expression of receptors and transporter proteins, as well as intracellular enzyme kinetics. Combination strategies that include pathway-specific inhibitors enhance potency but raise considerations about off-target effects and drug–drug interactions. So the, nano-phytomedicines offer precisely controlled modulation of PI3K/Akt, MAPK, and NF-κB pathways via improved delivery and targeting, translating phytochemical bioactivity into potent therapeutic interventions [123]. Table 3 illustrates the multifaceted biological actions mediated by nano-phytomedicine systems, emphasizing their capacity to orchestrate intracellular processes beyond conventional pharmacology. A critical observation is the convergence of these mechanisms toward restoring homeostasis in diseased cells be it through oxidative balance, gene expression modulation, or signaling pathway correction. Importantly, nano-enabled delivery allows precise spatial and temporal control over bioactivity, which is vital when modulating interconnected cellular circuits. For example, regulating redox states through Nrf2 not only suppresses oxidative damage but also affects downstream epigenetic and apoptotic events. Likewise, manipulating epigenetic regulators like DNMTs or HDACs via nanoformulations offers the potential for long-term reprogramming of pathological gene networks a feature highly desirable in chronic diseases or cancer prevention. Additionally, the synergy among various pathways such as simultaneous suppression of PI3K/Akt and NF-κB underscores the systems-level impact nano-phytochemicals can exert. Collectively, this table reflects how nanotechnology transforms phytochemicals into multitargeted therapeutic platforms.

### 4.5. Epigenetic and Genomic Regulation by Nano-Phytochemicals

Emerging evidence suggests that nano-phytomedicines can regulate gene expression and epigenetic markers, adding another layer to their therapeutic impact. Phytochemicals like curcumin, resveratrol, and sulforaphane exhibit epigenetic actions such as DNA methylation inhibition, histone modification, and microRNA regulation which can be augmented when delivered via nanocarriers [124]. DNA methyltransferases (DNMTs) maintain gene-silencing methyl marks in promoters of tumor suppressor genes. Phytochemicals such as EGCG and sulforaphane are natural DNMT inhibitors. Nanoformulations improve nuclear delivery, increasing intracellular concentration and nuclear accumulation. For instance, PLGA nanoparticles loaded with EGCG reduce DNMT activity and promote re-expression of silenced tumor suppressor genes in prostate cancer cell lines [125]. Sustained-release kinetics help maintain anti-DNMT activity with fewer dosing frequency limitations. Histone acetylation status regulates DNA accessibility. Phytochemicals like curcumin act as histone deacetylase (HDAC) inhibitors, increasing histone acetylation and promoting gene transcription. Enhanced delivery via nano-carriers ensures effective nuclear concentration. Curcumin-loaded nanocarriers have shown greater HDAC inhibition and reactivation of silenced genes in leukemia models compared to free drug [126]. MicroRNAs (miRNAs) regulate multiple genes and pathways simultaneously. Nano-phytomedicines can influence miRNA expression by improving cellular uptake. Resveratrol-loaded polymeric nanoparticles can upregulate tumor-suppressive miRNAs such as miR-34a and downregulate oncogenic miR-21 in cancer cells, leading to reduced proliferation and increased apoptosis [127]. Epigenetic reprogramming via nano-phytomedicines has lasting effects on gene expression. For example, early exposure to curcumin-loaded nanoparticles induces chromatin state alterations in stem cell models, influencing differentiation pathways and disease resistance [128]. These findings highlight the potential of nano-phytomedicines in disease prevention, beyond treatment. Epigenetic modulation affects broad gene networks, raising concerns about unintended gene activation. Controlled release systems help achieve therapeutic concentrations while avoiding epigenetic overstimulation. Tissue-specific targeting assists in avoiding off-target gene regulation. Safety and efficacy evaluation must include genome-wide expression profiling. Nano-phytomedicines’ ability to modulate cellular signaling and epigenetic landscapes adds depth to their pharmacological potential. By providing enhanced delivery to intracellular and nuclear targets, these systems drive therapeutic effects beyond conventional mechanisms, enabling precise and durable regulation of disease-relevant pathways [129].

**Figure 4:**
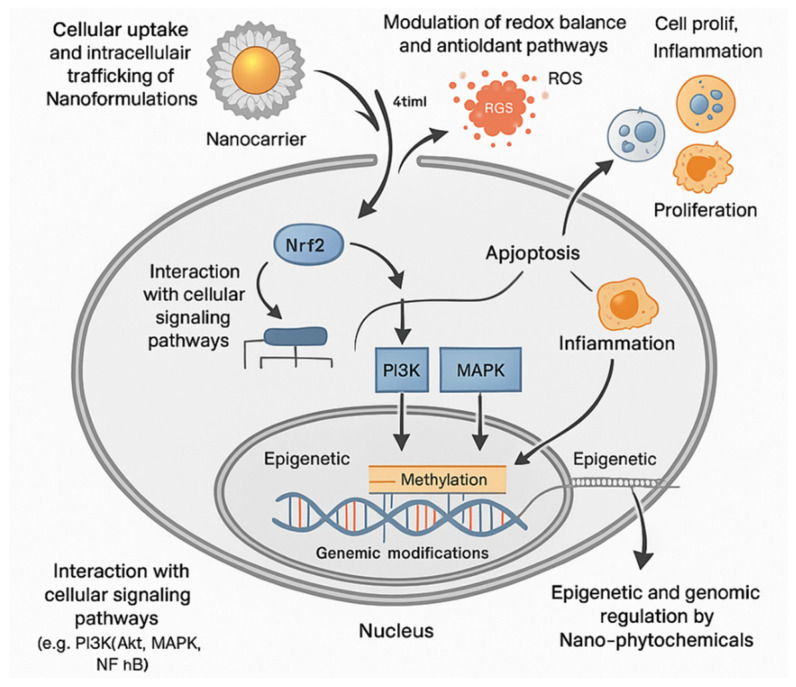
Schematic of the molecular mechanisms of action of nano-phytomedicine: cellular uptake, redox modulation, signaling interference, and epigenetic regulation.

Figure 4 illustrates the multifaceted molecular mechanisms by which nano-phytomedicines exert their therapeutic effects at the cellular level. Upon administration, nanocarriers facilitate the efficient uptake of encapsulated phytochemicals through endocytosis, often via clathrin- or caveolae-mediated pathways, enabling intracellular trafficking toward endosomes and lysosomes. Once internalized, these nanoformulations modulate oxidative stress by scavenging reactive oxygen species (ROS) and activating the Nrf2 pathway, thereby restoring redox homeostasis and preventing cellular damage. Concurrently, nano-phytochemicals influence cell fate by regulating key apoptotic and proliferative markers, including caspases, Bcl-2 family proteins, and pro-inflammatory cytokines, resulting in anti-inflammatory and anticancer effects. Additionally, these formulations interact with critical signaling cascades such as PI3K/Akt, MAPK, and NF-κB, inhibiting pathological cell survival and inflammatory responses. Importantly, nano-phytomedicines also impact epigenetic and genomic processes by altering DNA methylation, histone modifications, and non-coding RNA expression, enabling long-term modulation of gene expression profiles. This comprehensive modulation underscores their potential as next-generation therapeutics for complex, chronic diseases.

## 5. Therapeutic Applications in Human Health

### 5.1. Cancer Therapy: Tumor Targeting and Chemoprevention

Nanocarrier-enabled delivery of phytochemicals offers a multifaceted approach to cancer therapy exemplifying enhanced targeting, controlled release, and synergistic mechanisms:

- **Improved tumor accumulation and retention**: Nanocarriers exploit the Enhanced Permeability and Retention (EPR) effect, resulting in higher tumor uptake compared to free compounds. Lipid- and polymer-based nanoformulations of curcumin, resveratrol, and EGCG have shown significantly increased tumor accumulation and therapeutic efficacy in breast, prostate, pancreatic, and colon cancers compared to their native forms [130,131].
- **Targeted delivery and cellular uptake**: Surface functionalization strategies—such as folate receptors, peptides, or antibodies—enhance selective cellular uptake. Folic acid-decorated lipid carriers loaded with resveratrol exhibited over twice the uptake in ovarian cancer cells versus controls [132]. Similarly, HER2-targeted polymeric nanoparticles delivering phytochemical paclitaxel precursors demonstrated potent cytotoxicity in breast cancer models [133].
- **Synergistic mechanisms in tumor suppression**: Phyto-nanocarriers modulate multiple anticancer pathways including apoptotic induction, cell cycle arrest, anti-angiogenesis, and ferroptosis. For example, carriers inducing ferroptosis—an iron-dependent form of cell death—have been shown to synergize with photodynamic therapies, leveraging ROS accumulation and GPX4 inhibition [134].
- **Clinical translation moving forward**: While numerous phyto-nanodrugs are undergoing preclinical evaluation, a subset has entered clinical testing or is nearing approval [135]. The AuroLase™ gold nanoshells (for head and neck, lung, and prostate tumors) exemplify how phytochemical-functionalized inorganic nanocarriers can be translated into the clinic [136].

These nanomedicine strategies transform natural products into precision oncology agents, with improved pharmacokinetics, targeting, and mechanistic diversity for chemoprevention and tumor treatment.

### 5.2. Cardiovascular Health: Anti-Atherogenic and Antihypertensive Effects

Phytochemical nanoformulations also show promise in the cardiovascular space, particularly in combating atherosclerosis and hypertension:

- **Anti-atherogenic potential**: Nanocarrier-delivered polyphenols (like resveratrol and catechins) and terpenoids mitigate oxidative stress and inflammation in endothelial cells. Enhanced bioavailability leads to reduced LDL oxidation, lower vascular smooth muscle proliferation, and decreased macrophage infiltration in atherosclerotic lesions [137,138]. Some systems leverage magnetic nanoparticles to localize phytochemicals to inflamed plaques under external guidance [139].
- **Blood pressure modulation**: Nanoformulations such as naringenin-loaded nanocarriers have demonstrated improved vasodilatory effects, enhanced endothelial nitric oxide synthesis, and reduced vascular resistance in hypertensive animal models. Compared to unencapsulated phytochemicals, these nanoformulations required lower doses for similar antihypertensive outcomes [140].
- **Enhanced therapeutic indices**: By increasing solubility and stability, nanocarriers enable sustained-release profiles, maintaining therapeutic concentrations over extended periods—crucial for managing chronic cardiovascular conditions [141].
- **Safety and translational outlook**: Lipid-based nanoemulsions and polymeric systems exhibit good biocompatibility and low toxicity in vivo. However, comprehensive long-term safety studies are needed. Clinical translational efforts are underway, though in vivo data and human trials are still limited [142].

Overall, nanoformulated phytochemicals offer a multifactorial therapeutic toolkit—antioxidant, anti-inflammatory, lipid-regulating, and antihypertensive—that may complement or improve upon current cardiovascular therapies.

### 5.3. Neurodegenerative Disorders: Blood–Brain Barrier Penetration and Neuroprotection 

Nanoformulated phytochemicals offer a promising solution for managing neurodegenerative diseases by enhancing delivery to the central nervous system (CNS), overcoming the restrictive blood–brain barrier (BBB):

- **Improved BBB permeability:** Traditional phytochemicals like curcumin and resveratrol face poor CNS bioavailability due to the semi-permeable BBB. Encapsulation in liposomes or polymeric nanoparticles coated with surfactants (e.g., polysorbate-80) or targeted ligands (e.g., transferrin) has enhanced brain uptake by several-fold in rodent models [143].
- **Neuroprotective effects:** Once inside the CNS, these nano-phytomedicines exert antioxidant activity, suppress neuroinflammation, and support mitochondrial function. For example, curcumin-loaded nanoparticles attenuated microglial activation, decreased levels of neurotoxic cytokines (TNF-α, IL-6), and improved cognitive performance in Alzheimer’s disease (AD) models [144].
- **Synergy with anti-aggregation mechanisms:** Nano-encapsulated resveratrol and epigallocatechin gallate (EGCG) interfere with amyloid-β aggregation and tau phosphorylation, key pathological events in neurodegeneration. Targeted delivery increased accumulation near amyloid plaques and enhanced reduction in fibril load [145].
- **Translational potential and safety:** While preclinical data are promising, translating these technologies into clinical use requires rigorous assessment of long-term toxicity, dose optimization, scalable production, and regulatory compliance for CNS-targeted nanotherapies [146].

### 5.4. Metabolic Disorders: Anti-Diabetic and Anti-Obesity Actions

Phytochemical-loaded nanocarriers play an increasingly significant role in managing metabolic diseases by addressing bioavailability issues and enabling targeted delivery:

- **Glycemic control and insulin sensitization:** Nanosystems encapsulating berberine, quercetin, and cinnamon polyphenols have shown improved intestinal absorption and bioactivity, leading to reduced fasting glucose, enhanced insulin sensitivity, and decreased HbA1c in diabetic animal models [147]. Silver and chitosan nanoparticles aid in increasing pancreatic β-cell survival and functionality through their controlled release characteristics [148].
- **Anti-obesity effects:** Nanoformulations that improve the bioavailability of curcumin or catechins have been shown to inhibit adipogenesis, bolster thermogenesis, and reduce fat accumulation in high-fat-diet rodent models [149]. Lipid polymer hybrids loaded with anti-obesity phytochemicals have achieved sustained release in adipose tissues, improving satiety hormone profiles and suppressing inflammatory cytokines [150].
- **Lipid profile modulation:** Nano-delivered silymarin and resveratrol have demonstrated significant reductions in LDL and triglycerides, with concurrent increases in HDL cholesterol [151]. Liposomal formulations appear to amplify the cholesterol-lowering mechanisms of phytochemicals.
- **Safety and therapeutic outcomes:** Nanocarrier-based approaches typically show good tolerability in preclinical studies; however, long-term analyses are necessary to verify metabolic safety and assess off-target effects in chronic administration settings [152].

Table 4 underscores the diverse and disease-specific therapeutic roles nano-phytomedicines can play when properly engineered and targeted. What stands out is the ability of these nanosystems not only to enhance solubility and stability of phytochemicals but also to modulate complex biological environments such as tumor microenvironments, atherosclerotic plaques, and inflamed neural tissue through spatially controlled delivery. A crucial advantage is their multifunctionality; for instance, many formulations exert both anti-inflammatory and antioxidant effects, which are central to diseases like neurodegeneration and diabetes, highlighting their system-level therapeutic potential. Moreover, the modular nature of nanocarriers permits combinatorial designs, enabling the co-delivery of multiple bioactives or the integration of imaging agents for theranostic applications. As nano-phytomedicine matures, these platforms are increasingly being tailored with stimuli-responsiveness such as pH-, enzyme-, or ROS-triggered release, offering greater precision and minimizing systemic side effects. This versatility places nano-phytomedicine at the forefront of next-generation, integrative, and personalized healthcare approaches.

### 5.5. Infectious Diseases: Antibacterial, Antiviral, and Antifungal Applications

The delivery of phytochemicals via nanocarriers is emerging as a powerful tool in combating infectious diseases:

- **Antibacterial strategies:** Phytochemical-loaded nanoparticles combine the intrinsic antimicrobial properties of metal (e.g., Ag, ZnO) or polymeric materials with the bioactivity of plant compounds. Silver nanoparticle–curcumin conjugates, for instance, exhibit synergistic bactericidal effects against MRSA and *E. coli*, disrupting biofilm formation and increasing bacterial membrane disruption [157].
- **Antiviral delivery systems:** Nano-encapsulation of quercetin, baicalin, or cynanoside enhances antiviral activity against influenza, herpes simplex, and coronaviruses. Phytochemical–gold nanoparticle conjugates block viral entry and replication via actions like ACE2 receptor inhibition or protease binding [153].
- **Antifungal efficacy:** Nanocapsules containing eugenol, thymol, and other essential oils maintain effective drug concentrations in target tissues, adding precision to fungal membrane disruption and inhibiting virulence factors [154].
- **Addressing antibiotic resistance:** These systems employ multifunctional mechanisms—ROS production, membrane interaction, efflux pump inhibition—delivering therapeutic synergy at lower, safer doses while reducing the potential for resistance development [93].
- **Stability and administration:** Encapsulation ensures controlled release, improved bioavailability, and enhanced topical or inhalable delivery, offering versatility in treating cutaneous, pulmonary, and systemic infections [155].

**Figure 5:**
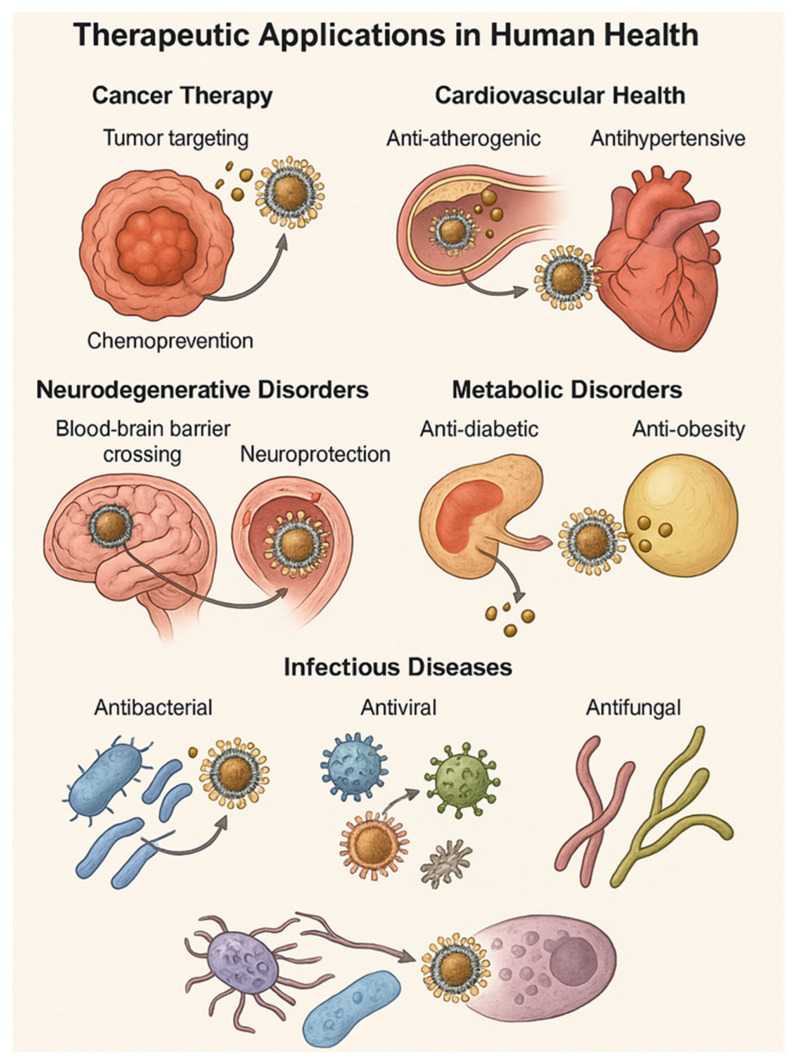
Therapeutic applications of nano-phytomedicine in human health.

Figure 5 illustrates the diverse therapeutic roles of nano-phytomedicines across major disease domains. Nanocarrier-based delivery systems enhance tumor targeting and chemoprevention in cancer therapy, exert anti-atherogenic and antihypertensive effects in cardiovascular diseases, enable blood–brain barrier penetration for neurodegenerative disorders, improve glycemic control and lipid metabolism in metabolic disorders, and offer potent antibacterial, antiviral, and antifungal actions in infectious disease management. The schematic highlights site-specific interactions that contribute to improved bioavailability and targeted therapeutic action.

## 6. Pharmacokinetics and Toxicological Considerations

### 6.1. Bioavailability and Controlled Release Kinetics

Phytochemicals often suffer from poor oral bioavailability due to intrinsic physicochemical limitations such as low aqueous solubility, poor permeability, and extensive first-pass metabolism. Nanocarrier systems have been extensively explored to overcome these barriers and enhance the pharmacokinetic profile of plant-derived bioactives [156]. The encapsulation of phytochemicals into nanoscale vehicles such as liposomes, polymeric nanoparticles, solid lipid nanoparticles (SLNs), and nanostructured lipid carriers (NLCs) has been demonstrated to significantly improve their solubility, protect against enzymatic degradation, and facilitate controlled and sustained release [158]. Controlled release kinetics is a critical parameter that dictates the therapeutic efficacy and safety profile of nano-phytomedicines. Conventional phytochemical formulations often show rapid clearance and short plasma half-life, limiting their clinical utility [159]. Nanocarriers can be engineered to provide zero-order or sustained release kinetics by manipulating polymer composition, particle size, surface chemistry, and encapsulation efficiency [160]. For instance, PLGA-based nanoparticles degrade hydrolytically, allowing predictable drug release profiles, which can be fine-tuned by adjusting polymer molecular weight and lactide/glycolide ratio [161]. Similarly, SLNs and NLCs leverage lipid matrix crystallinity and composition to control the diffusion of phytochemicals, ensuring prolonged systemic circulation and sustained therapeutic levels [162]. The improved bioavailability conferred by nanocarriers is often attributed to multiple synergistic factors: enhanced dissolution rates due to nanometric size and increased surface area; protection from acidic and enzymatic degradation in the gastrointestinal tract; and facilitated transport across epithelial barriers via endocytosis or paracellular routes [163]. In particular, mucoadhesive polymers like chitosan can transiently open tight junctions, improving paracellular permeability of phytochemicals such as curcumin and quercetin [164]. Pharmacokinetic studies have repeatedly demonstrated that nano-encapsulated phytochemicals achieve higher maximum plasma concentrations (C-max), longer time to reach peak concentration (T-max), and increased area under the curve (AUC) compared to free compounds [165]. For example, liposomal curcumin exhibited a 4- to 5-fold increase in oral bioavailability relative to free curcumin in rodent models [166]. Controlled release formulations also reduce dosing frequency and minimize plasma level fluctuations, enhancing patient compliance and therapeutic outcomes [167]. However, alongside pharmacokinetic enhancements, regulatory scrutiny is increasing regarding excipient safety, nanocarrier degradation products, and release kinetics predictability. Lack of harmonized regulatory guidelines for herbal nanomedicine adds complexity to clinical translation. Formulators must address issues such as reproducibility of release profiles across batches and scale and provide robust in vitro–in vivo correlation (IVIVC) models to gain regulatory acceptance. Controlled release kinetics also pose challenges. Burst release rapid initial release of a significant fraction of the payload may cause toxicity or reduced therapeutic duration [168]. Formulation optimization requires balancing drug loading, carrier stability, and release mechanisms to achieve desired pharmacokinetics. Stimuli-responsive nanocarriers, which release phytochemicals in response to pH, enzymes, temperature, or redox conditions, are promising to further refine delivery precision [169]. In conclusion, nanocarrier-mediated improvements in bioavailability and controlled release kinetics represent a cornerstone of modern phytochemical therapeutics. Nonetheless, for regulatory approval, developers must also meet safety standards related to excipient toxicity, batch consistency, and documentation of long-term stability. Rigorous in vitro–in vivo correlation studies and pharmacokinetic modeling remain essential to optimize formulations for clinical translation, ensuring maximized efficacy with minimized adverse effects [170].

### 6.2. In Vivo Biodistribution and Organ Targeting

**Figure 6:**
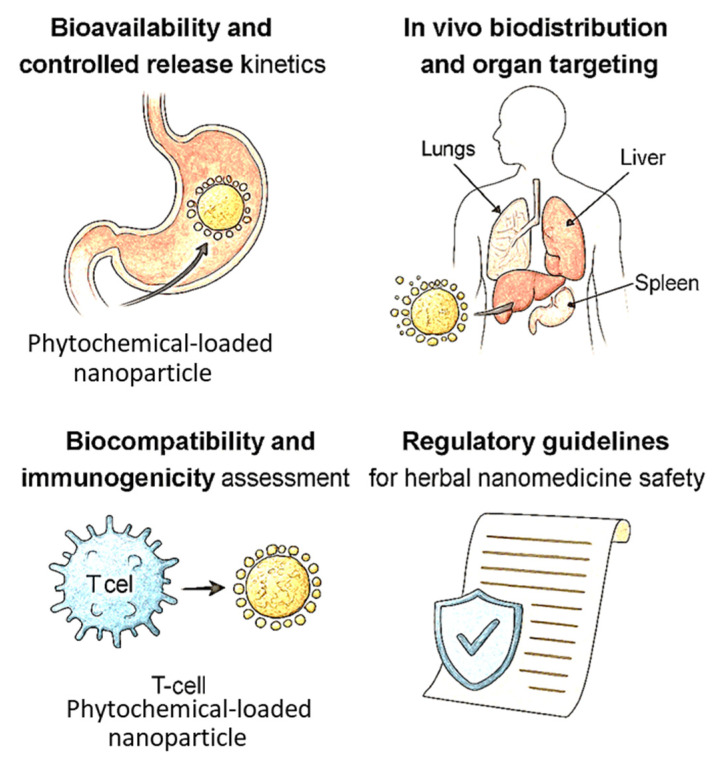
Pharmacokinetic and toxicological framework of nano-phytomedicine.

Understanding the in vivo biodistribution of phytochemical-loaded nanocarriers is fundamental to optimizing therapeutic efficacy and minimizing off-target toxicity. Biodistribution studies elucidate how nanoparticles distribute across organs and tissues after administration, shedding light on their pharmacokinetic profiles, clearance mechanisms, and accumulation at target sites. Effective organ targeting enhances drug concentration at disease sites while sparing healthy tissues, thus improving the therapeutic index of nano-phytomedicines [171]. Nanocarriers administered via different routes (oral, intravenous, topical, or pulmonary) exhibit distinct biodistribution patterns governed by size, shape, surface charge, and chemical composition. Generally, nanoparticles sized between 10 and 200 nm achieve optimal circulation time and reduced renal clearance, permitting enhanced extravasation into tissues with compromised vasculature, such as tumors and inflamed sites, through the enhanced permeability and retention (EPR) effect [172]. However, passive targeting via EPR alone is often insufficient for selective delivery, especially in diseases where pathological vasculature heterogeneity limits nanoparticle access [173]. Surface modification with polyethylene glycol (PEG) and other hydrophilic polymers (PEGylation) is widely employed to confer “stealth” properties, reducing protein adsorption (opsonization) and subsequent clearance by the mononuclear phagocyte system (MPS), predominantly in liver and spleen [174]. This prolongs systemic circulation and enhances accumulation at the target site [175]. PEGylated phytochemical nanocarriers have demonstrated increased plasma half-life and improved therapeutic outcomes in preclinical cancer and inflammatory models [176]. Active targeting strategies further enhance biodistribution specificity by decorating nanocarrier surfaces with ligands that bind to receptors overexpressed on diseased cells or tissues. Common targeting moieties include antibodies, peptides, aptamers, and small molecules such as folic acid [177]. For example, folate receptor-targeted nanocarriers delivering resveratrol have shown significantly greater uptake in ovarian cancer tissues compared to non-targeted systems, resulting in improved tumor regression [178]. Similarly, transferrin-modified nanoparticles facilitate efficient blood–brain barrier penetration for neuroprotective phytochemicals [179]. Nanocarrier physicochemical properties also influence biodistribution. Positively charged nanoparticles tend to interact more readily with negatively charged cell membranes but often exhibit rapid clearance and increased toxicity, while neutral or slightly negative surface charges balance circulation time and cellular uptake [180]. Shape and rigidity also affect biodistribution: rod-shaped or flexible particles show enhanced margination and tumor penetration compared to rigid spheres [181]. Organ-specific delivery approaches have been explored to concentrate phytochemicals in specific tissues. For instance, magnetic nanoparticles conjugated with phytochemicals enable magnetic field-guided targeting to tumor or inflamed sites, reducing systemic exposure and toxicity [182]. Similarly, pulmonary delivery of phytochemical-loaded nanoparticles achieves high local concentrations in the lungs for respiratory diseases, bypassing systemic distribution [183]. From a regulatory perspective, demonstrating consistent and reproducible biodistribution profiles across preclinical models is critical for approval. Safety concerns include unintended accumulation in off-target organs such as the liver, kidneys, and spleen, and potential for chronic toxicity or immune activation. Standardized imaging and biodistribution protocols are needed to meet agency expectations for pharmacokinetic validation. Imaging modalities such as fluorescence, MRI, PET, and CT coupled with labeled nanoparticles provide quantitative biodistribution data, enabling real-time monitoring and optimization of delivery systems [184]. These studies have revealed organ accumulation patterns, clearance routes (renal vs. hepatobiliary), and off-target accumulation risks, informing rational nanocarrier design [185]. However, challenges remain in achieving precise targeting, avoiding uptake by off-target organs (e.g., liver, spleen), and translating biodistribution data from animal models to humans due to interspecies physiological differences. Understanding the protein corona formation on nanoparticles in biological fluids is also critical, as it influences biodistribution and cellular interactions [186]. Furthermore, lack of international standardization in labeling methods, imaging endpoints, and biodistribution metrics complicate regulatory harmonization. In summary, in vivo biodistribution and organ targeting govern the success of phytochemical nanocarriers. Integration of passive and active targeting strategies, surface engineering, and advanced imaging techniques enable enhanced therapeutic outcomes with reduced side effects, paving the way for precision nanomedicine applications [187]. Figure 6 encapsulates the integrated pharmacokinetic and toxicological determinants that critically influence the therapeutic efficacy and clinical viability of nano-phytomedicines. Nanocarriers significantly enhance bioavailability by improving the solubility and stability of poorly soluble phytochemicals and enabling controlled and sustained release profiles that mitigate rapid clearance and dosing frequency. The figure emphasizes how strategic nanocarrier design facilitates targeted biodistribution, allowing selective accumulation in diseased tissues such as tumors or inflamed sites while minimizing systemic toxicity. Surface functionalization with ligands or stealth polymers like PEG extends circulation time and improves site-specific uptake, a pivotal factor in achieving therapeutic precision. Moreover, the biocompatibility and immunogenicity assessment segment underlines the need for comprehensive safety profiling, including cytotoxicity, hemocompatibility, and immune system interactions, which are vital to prevent adverse responses. Finally, the figure integrates regulatory considerations, acknowledging the necessity of compliance with international safety guidelines and standardized evaluation protocols to support clinical translation. Together, these elements underscore the multidimensional approach required to develop safe, effective, and regulatory-compliant nano-phytomedicines.

### 6.3. Biocompatibility and Immunogenicity Assessment

The biocompatibility and immunogenicity of nanocarriers encapsulating phytochemicals are pivotal determinants of their clinical viability and safety. Nanomaterials introduced into the body interact with complex biological systems, potentially triggering immune responses or cytotoxic effects that may limit therapeutic utility or cause adverse reactions [188]. Therefore, rigorous biocompatibility assessment and immunogenicity profiling form integral components of the preclinical evaluation of nano-phytomedicines. Biocompatibility refers to the ability of nanocarriers to perform their desired function without eliciting harmful local or systemic effects. This encompasses cytotoxicity, genotoxicity, hemocompatibility, and effects on normal tissue architecture and function. In vitro cytotoxicity assays using relevant cell lines (e.g., hepatocytes, renal tubular cells, endothelial cells) provide initial screening for nanoparticle safety. Many nanocarriers composed of biodegradable polymers (e.g., PLGA, chitosan) and lipids exhibit low inherent cytotoxicity, attributed to their biocompatible degradation products [189]. For instance, chitosan-based nanoparticles show favorable cellular uptake with minimal cytotoxicity at therapeutic concentrations [190]. Immunogenicity, the ability of nanomaterials to provoke immune activation, is a complex phenomenon involving recognition by the innate immune system, complement activation, cytokine release, and adaptive immune responses. Nanocarriers may be recognized as foreign entities, leading to opsonization, clearance by macrophages, and potential hypersensitivity reactions [191]. The physicochemical characteristics of nanoparticles—size, shape, surface charge, and chemistry critically influence immunogenicity. For example, positively charged nanoparticles often induce stronger immune activation compared to neutral or negatively charged particles [192]. Surface modifications such as PEGylation have been widely adopted to reduce immunogenicity by creating a hydrophilic “stealth” layer that minimizes protein adsorption and macrophage uptake [193]. However, repeated administration of PEGylated nanocarriers can induce anti-PEG antibodies, resulting in accelerated blood clearance and reduced efficacy, a phenomenon known as the “accelerated blood clearance” (ABC) effect [194]. Alternatives such as zwitterionic polymers or biomimetic coatings (e.g., cell membrane camouflage) are under investigation to overcome these limitations [195]. Hemocompatibility assessment evaluates the interaction of nanoparticles with blood components, including erythrocytes, platelets, and coagulation factors. Nanocarriers should not induce hemolysis, platelet aggregation, or coagulation cascade activation to avoid thrombosis or bleeding risks [196]. Several studies report that lipid-based and polymeric phytochemical nanocarriers maintain good hemocompatibility profiles, but careful evaluation is essential for each formulation [197]. In vivo immunotoxicity studies involve monitoring cytokine profiles (e.g., TNF-α, IL-6, IL-1β), complement activation, and histopathological changes in immune organs (spleen, lymph nodes). Animal models reveal that some inorganic nanoparticles (e.g., metal oxides) can induce oxidative stress-mediated immune activation, necessitating dose optimization and surface functionalization [198]. Long-term toxicity studies are indispensable for chronic use phytomedicines, as accumulation or degradation products may cause delayed adverse effects. Nanocarrier biodegradation rates, metabolite toxicity, and potential accumulation in reticuloendothelial system organs require thorough investigation [199]. Regulatory agencies emphasize robust biocompatibility and immunogenicity data in nanomedicine approval pathways. International standards (e.g., ISO 10993) [200] guide preclinical testing frameworks, including in vitro assays, animal studies, and clinical monitoring protocols [201]. Employing standardized methodologies and relevant models ensures reproducibility and safety benchmarking across studies. So the biocompatibility and immunogenicity assessments are crucial to balancing therapeutic benefits and safety risks in nano-phytomedicine development. Rational nanocarrier design, including surface engineering and choice of biocompatible materials, alongside comprehensive preclinical and clinical evaluation, underpins the successful translation of phytochemical nanotherapies to the clinic [202,203].

Table 5 presents a multidimensional perspective on how nanoscale engineering profoundly reshapes the pharmacokinetic behavior and toxicological safety of phytochemical-based therapies. Beyond mere enhancement of solubility or protection from degradation, nanocarriers serve as dynamic modulators of biological interactions governing absorption routes, systemic retention, immune recognition, and metabolic fate. Notably, the strategic manipulation of surface chemistry (e.g., PEGylation, zwitterionic coatings) and morphology (e.g., core-shell vs. matrix) allows for tailored biodistribution and controlled release, aligning therapeutic delivery with pathophysiological needs. However, this sophistication introduces complexity in predicting in vivo responses, as subtle changes in charge, shape, or protein corona formation can shift organotropism or immune compatibility. The integration of nanocarrier adaptability with regulatory-standardized safety assessment, as reflected in ISO-guided protocols, ensures translational viability. In essence, Table 5 underscores that effective nano-phytomedicine design demands not only bioavailability enhancement but a harmonized balance between efficacy, biocompatibility, and immunological invisibility.

### 6.4. Regulatory Guidelines for Herbal Nanomedicine Safety

The clinical translation of herbal nanomedicines, including phytochemical-loaded nanocarriers, necessitates strict adherence to regulatory frameworks that ensure safety, efficacy, and quality. Regulatory agencies worldwide, such as the U.S. Food and Drug Administration (FDA), European Medicines Agency (EMA), and others, have developed evolving guidelines that address the unique challenges posed by nanoscale drug delivery systems, especially those derived from herbal sources [204]. One primary challenge is the inherent complexity of phytochemical extracts, which contain multiple bioactive constituents with variable compositions influenced by botanical source, extraction methods, and batch variations [205]. When these extracts are integrated into nanocarriers, characterization becomes more intricate due to the interplay of nanomaterial properties and herbal matrix complexity. Regulators require comprehensive physicochemical characterization including particle size distribution, surface charge, morphology, chemical composition, and stability to ensure reproducibility and batch consistency [206]. Toxicological evaluation remains paramount. Herbal nanomedicines must undergo rigorous in vitro and in vivo toxicology assessments, including genotoxicity, cytotoxicity, immunotoxicity, and long-term carcinogenicity studies tailored for both the phytochemical components and the nanocarrier materials [207]. The potential for synergistic toxicity arising from nanoparticle–phytochemical interactions necessitates specific investigation [208]. Regulatory guidance encourages the use of standardized testing protocols such as ISO 10993 and OECD Test Guidelines, adapted for nanoformulations [209]. Pharmacokinetic and pharmacodynamic profiling must demonstrate improved bioavailability and therapeutic benefit without compromising safety. Regulatory agencies stress the importance of detailed ADME (absorption, distribution, metabolism, and excretion) studies and the identification of potential toxic metabolites arising from the phytochemical or nanomaterial degradation [210]. Special attention is given to off-target accumulation and long-term retention in organs such as liver, spleen, kidneys, and brain [211]. Clinical trial design for herbal nanomedicines should include safety endpoints specific to nano-bio interactions, immunogenicity monitoring, and post-market surveillance plans [212]. Given the variable regulatory landscapes for botanical drugs and nanomedicines, hybrid regulatory pathways are emerging. For example, the FDA’s Botanical Drug Development Guidance provides a framework for complex herbal products, which can be combined with the agency’s guidance on nanotechnology applications [213]. Quality control is another critical focus. Current Good Manufacturing Practices (cGMP) must be adapted to accommodate nanoscale materials, including control of nanoparticle size, distribution, surface properties, and loading efficiency. Analytical methods like dynamic light scattering (DLS), electron microscopy, high-performance liquid chromatography (HPLC), and mass spectrometry are essential for routine quality assurance [214]. International harmonization efforts are underway to standardize definitions, characterization methods, and safety assessment protocols for herbal nanomedicines. Organizations such as the International Council for Harmonisation (ICH) and ISO play significant roles in developing guidelines that balance innovation and patient safety [215]. Despite these advances, regulatory approval of herbal nanomedicines remains challenging due to the novelty of the field, complexities in standardization, and limited long-term clinical safety data. Collaboration among researchers, industry, and regulators is vital to establish robust frameworks that facilitate safe and effective commercialization of nano-phytomedicines [216]. In summary, regulatory guidelines for herbal nanomedicine safety are evolving to address the complexities of combining botanical therapeutics with nanotechnology. Comprehensive physicochemical, toxicological, pharmacokinetic, and clinical evaluations aligned with international standards are essential to ensure that these innovative therapies reach patients safely and effectively [217].

## 7. Clinical Advancements and Translational Challenges

### 7.1. Preclinical Studies and Clinical Trials of Nano-Phytomedicines

The journey from bench to bedside for nano-phytomedicines is grounded in extensive preclinical evaluations, followed by carefully designed clinical trials. Preclinical studies provide foundational insights into the efficacy, pharmacokinetics, biodistribution, toxicity, and mechanisms of action of phytochemical-loaded nanocarriers. Numerous in vitro and in vivo models have demonstrated enhanced therapeutic outcomes when phytochemicals are delivered via nanocarriers compared to free compounds. For example, curcumin-loaded polymeric nanoparticles have exhibited superior anti-inflammatory and anticancer activities in murine models by improving bioavailability and targeting inflammatory pathways [218]. Rodent and non-rodent animal models play an indispensable role in evaluating the safety profiles and therapeutic indices of nano-phytomedicines. Toxicology studies assess acute, subacute, and chronic effects, including immune responses, organ-specific toxicity, and genotoxicity, informing safe dosage ranges for human trials [219]. Pharmacokinetic studies using techniques such as LC-MS/MS and imaging modalities provide quantitative data on absorption, distribution, metabolism, and excretion [220]. Furthermore, advanced models that better mimic human pathophysiology, such as patient-derived xenografts or organ-on-chip systems, are increasingly employed to bridge translational gaps [221]. Despite promising preclinical data, nano-phytomedicines face hurdles in clinical trial advancement. Clinical trials are limited, largely due to regulatory complexities, scale-up challenges, and the need for robust safety data. Regulatory bodies often lack harmonized frameworks specifically tailored to botanical-based nanomedicines, creating ambiguity in classification—whether as pharmaceuticals, nutraceuticals, or supplements. This ambiguity delays approval pathways, especially when phytochemical compositions vary due to source differences, seasonal variation, and extraction methods. Moreover, the inherently complex, multi-component nature of botanical extracts presents challenges in defining active pharmaceutical ingredients (APIs), which is a fundamental requirement for regulatory clearance [222]. Some formulations have reached Phase I and II trials, primarily focusing on cancer and inflammatory diseases. For instance, a liposomal curcumin formulation has undergone Phase II trials for pancreatic cancer, demonstrating safety and preliminary efficacy [222]. Another example is a solid lipid nanoparticle formulation of resveratrol under investigation for metabolic syndrome management [223]. However, achieving reproducibility of these formulations on a commercial scale remains difficult. Variability in plant sources, differences in phytochemical profiles, and diverse manufacturing techniques contribute to batch-to-batch inconsistency. This not only complicates pharmacokinetic and pharmacodynamic profiling but also undermines regulatory confidence in product stability and uniformity [224]. Standardization challenges further compound translational hurdles. Unlike synthetic drugs with a single active compound, phytochemicals often consist of multiple synergistic components, making it difficult to standardize identity, purity, and potency. Robust and validated analytical methods are essential to ensure consistent phytochemical loading and release profiles, yet such methods are still evolving for complex botanical nanocarriers [225]. Additionally, ensuring appropriate characterization of nanoformulation parameters such as particle size, surface charge, encapsulation efficiency, and release kinetics is critical for regulatory submissions but remains underreported or poorly controlled in some studies. Patient variability, including differences in metabolism, immune status, and comorbidities, further complicates clinical trial design and outcome interpretation [225]. Ultimately, preclinical models have robustly demonstrated the therapeutic promise of nano-phytomedicines, but the transition to human trials requires overcoming substantial scientific, regulatory, and manufacturing barriers. Continued development of predictive preclinical platforms, alongside adaptive clinical trial designs, will be crucial to accelerate the clinical translation of these promising therapeutics [226].

### 7.2. Commercial Products and Patent Landscape

The commercialization of nano-phytomedicines is still nascent but growing steadily, with several products and patents highlighting the field’s progress and intellectual property (IP) dynamics. Commercial products combining phytochemicals with nanocarriers predominantly target areas such as oncology, dermatology, metabolic disorders, and wound healing [227]. Several liposomal, polymeric, and solid lipid nanoparticle-based phytochemical formulations have entered the market, often marketed as dietary supplements or cosmeceuticals with enhanced bioavailability claims. For example, liposomal curcumin and resveratrol products advertise improved absorption and therapeutic potential for general wellness and anti-inflammatory support [228]. However, few nano-phytomedicines have achieved full pharmaceutical regulatory approval due to stringent safety and efficacy requirements. The patent landscape reflects intense innovation activity, with patents covering novel nanocarrier compositions, surface functionalization methods, targeted delivery systems, and controlled release mechanisms. Patent filings from academia and industry reveal focus areas such as multifunctional hybrid nanocarriers, stimuli-responsive release, and combination therapies integrating phytochemicals with conventional drugs [229]. For instance, patents protecting curcumin-loaded polymeric nanoparticles with tumor-targeting ligands have been granted in multiple jurisdictions [230]. However, the multi-component nature of plant-derived extracts introduces unique challenges for IP protection. Unlike synthetic single-molecule drugs, botanical formulations often consist of complex mixtures of bioactive and non-bioactive components that can vary depending on the source, extraction method, and seasonality. This complexity complicates the standardization of formulations and makes it difficult to claim ownership over the exact composition, leading to reliance on process patents or delivery system patents instead of compound-specific protection [231]. Moreover, existing patent frameworks may not sufficiently accommodate the traditional and empirical knowledge embedded in phytomedicine, requiring innovative legal strategies to establish novelty and inventive steps. This can impact the strength and scope of patent protection, potentially shortening the exclusivity period and reducing incentives for commercial investment. In particular, patent examiners may question the reproducibility and stability of phytochemical compositions if variations between batches are not well characterized, creating hurdles during prosecution. Regulatory definitions also impact market entry. Herbal products are often regulated as supplements or traditional medicines, while nanoformulations with therapeutic claims may be classified as drugs, subjecting them to more rigorous approval pathways [232]. This regulatory dichotomy adds another layer of complexity for manufacturers. A single nano-phytomedicine product may fall under different regulatory frameworks depending on jurisdiction, dosage, route of administration, or labeling claims. Companies must often choose between faster but limited supplement registration and slower, more stringent drug approval routes each with distinct implications for clinical evidence requirements, marketing, and labeling. Furthermore, evolving guidelines for nanomedicines complicate submission strategies, as regulators may demand detailed characterization data on nanocarrier composition, stability, and interaction with phytochemicals. Companies must navigate this dual regulatory landscape to successfully commercialize products. Despite challenges, the growing consumer demand for natural and nanotechnology-enhanced health products, coupled with advances in nanomanufacturing and formulation science, is likely to drive expansion in the nano-phytomedicine market [233]. Partnerships between academia, biotech startups, and pharmaceutical companies facilitate translation from patent innovation to market-ready products.

**Figure 7:**
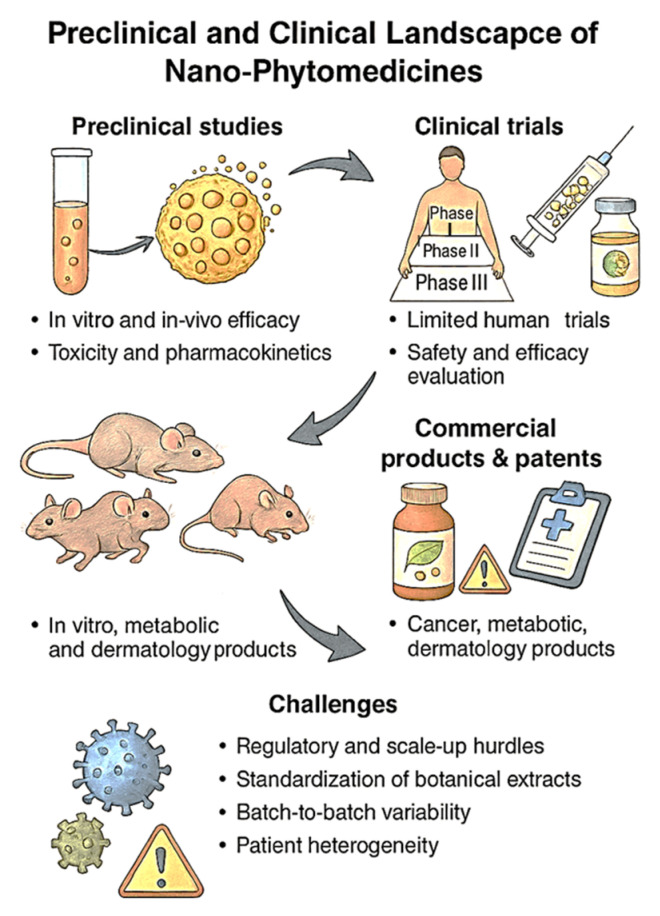
Schematic overview of nano-phytomedicine pharmacology highlighting bioavailability, biodistribution, biocompatibility, and regulatory safety considerations.

Figure 7 provides a comprehensive schematic representation of the core pharmacokinetic and toxicological parameters that influence the clinical translation of nano-phytomedicines. The figure illustrates how nanocarrier-based delivery systems significantly enhance the bioavailability of phytochemicals by facilitating controlled and sustained release, improving solubility, and protecting the bioactives from enzymatic degradation. Additionally, it highlights targeted organ biodistribution achieved through passive mechanisms like the enhanced permeability and retention (EPR) effect, as well as active targeting strategies involving ligand-functionalized nanocarriers. The integration of biocompatible materials and surface modifications such as PEGylation contributes to reduced immunogenicity and systemic toxicity, which are critical for long-term therapeutic safety. Furthermore, the figure underscores the importance of preclinical safety assessment frameworks aligned with regulatory guidelines to ensure consistency, reproducibility, and risk mitigation in herbal nanomedicine development. This visual encapsulation reinforces the multifaceted role of nanotechnology in optimizing phytochemical pharmacology for clinical applications.

### 7.3. Scale-Up and Manufacturing Barriers

Translating nano-phytomedicines from laboratory-scale prototypes to industrial-scale manufacturing presents several formidable challenges. Achieving reproducible, scalable, and cost-effective production of nanocarriers encapsulating complex phytochemical mixtures requires addressing formulation, process engineering, and quality control issues [234]. Batch-to-batch consistency is critical for regulatory approval and therapeutic reliability. Variability in raw botanical material composition can affect nanoparticle loading efficiency, stability, and bioactivity. Standardized sourcing, extraction, and quality assessment of phytochemical inputs are prerequisites for manufacturing consistency [235]. One of the most critical bottlenecks lies in the intrinsic heterogeneity of plant-derived raw materials, which exhibit seasonal, geographical, and species-related variability. This directly impacts the reproducibility of nanoparticle characteristics such as size, charge, drug loading capacity, and release profile—parameters essential for therapeutic efficacy and regulatory compliance. Manufacturing processes such as solvent evaporation, nanoprecipitation, high-pressure homogenization, and microfluidics must be optimized for scale without compromising particle size distribution, surface properties, and encapsulation efficiency [236]. Microfluidic systems show promise for continuous, scalable production with precise control over particle characteristics, but integration into industrial workflows remains limited [237]. Sterility, endotoxin control, and removal of residual solvents or surfactants are additional manufacturing hurdles, especially for parenteral formulations [238]. Robust purification and aseptic processing are mandatory, increasing complexity and cost. High-throughput purification, filtration, and post-synthesis stabilization processes often require specialized infrastructure not available in standard pharmaceutical plants, further slowing industrial adoption. Additionally, the cost of scaling these methods particularly when dealing with sensitive and multicomponent phytochemical payloads remains a formidable economic barrier. Analytical characterization methods need adaptation for high-throughput environments, enabling rapid assessment of nanoparticle size, polydispersity, surface charge, and payload content [239]. Process analytical technologies (PAT) can facilitate real-time monitoring and control, supporting quality by design (QbD) principles [240]. Regulatory expectations emphasize GMP compliance, documentation, and validation of manufacturing protocols. Facilities require significant capital investment and specialized expertise in nanotechnology production [241]. Infrastructural deficiencies, especially in regions lacking established nanomanufacturing ecosystems, impede technology transfer and localization of nano-phytomedicine production. Moreover, interdisciplinary knowledge gaps between natural product chemists and nanofabrication engineers exacerbate process inefficiencies. Moreover, the environmental impact of nanoparticle manufacturing, including solvent use and waste disposal, calls for green chemistry and sustainable manufacturing practices [242]. In sum, overcoming scale-up and manufacturing barriers involves a multidisciplinary approach integrating materials science, process engineering, quality control, and regulatory compliance. Collaborative efforts and technological innovations are essential to bring nano-phytomedicines to large-scale production and market availability [243].

### 7.4. Cost, Accessibility, and Acceptance in Healthcare Systems

Cost-effectiveness, accessibility, and acceptance within healthcare systems are critical factors influencing the successful adoption of nano-phytomedicines. Although nanotechnology can improve phytochemical therapeutic profiles, the added complexity and manufacturing demands often increase production costs compared to traditional formulations [244]. Economic evaluations must consider not only manufacturing expenses but also potential healthcare savings through improved efficacy, reduced side effects, and decreased dosing frequency [245]. Cost–benefit analyses that incorporate quality-adjusted life years (QALYs) and real-world patient outcomes will guide reimbursement and formulary decisions. Beyond direct costs, hidden economic burdens such as supply chain gaps, import tariffs on nanomaterials, and limited reimbursement policies for natural product-based therapies can significantly hinder clinical integration, particularly in resource-constrained settings. Accessibility in low- and middle-income countries may be hindered by infrastructure limitations, lack of technical expertise, and regulatory disparities. Simplifying formulation processes and leveraging scalable manufacturing technologies could enhance global accessibility [246]. However, widespread clinical deployment is further hampered by sociocultural and systemic challenges, including mistrust in novel technologies, skepticism toward herbal medicines in technologically advanced healthcare settings, and insufficient training of healthcare professionals in nanomedicine. These factors can reduce prescribing confidence and impact adoption rates. Healthcare professional and patient acceptance depend on demonstrated clinical benefits, safety, and clear communication regarding nanotechnology’s advantages and risks. Concerns about nanoparticle toxicity or unfamiliarity with nanomedicine can influence prescribing behavior and patient compliance [247]. Education and awareness programs targeting clinicians, pharmacists, and patients are necessary to build trust and understanding. Equally important is the integration of nano-phytomedicine education into medical and pharmaceutical curricula to equip future practitioners with the knowledge required to evaluate and apply these technologies effectively. Without such systemic educational reform, the innovation-to-application gap may persist. Integration into evidence-based guidelines and endorsement by professional societies will further facilitate acceptance [248]. Lastly, ethical considerations around equitable access and affordability must be addressed to prevent disparities in nano-phytomedicine utilization [249]. Public–private partnerships that support local manufacturing, reduce dependency on imports, and subsidize development costs for essential nano-phytomedicines can improve affordability. Simultaneously, equitable licensing and access frameworks must be promoted to prevent health inequality. Strategic collaborations among stakeholders, including governments, industry, and academia, are essential to navigate cost and acceptance challenges and realize the full potential of nano-phytomedicines in healthcare systems worldwide [250]. Table 6 illustrates the multidimensional progression of nano-phytomedicine from laboratory research to real-world healthcare implementation, highlighting how each stage of development presents both promise and complexity. While preclinical data have validated the potential of nanocarriers to improve phytochemical efficacy and pharmacokinetics, transitioning to clinical success demands more than therapeutic performance—it requires regulatory foresight, formulation standardization, and predictive translational models. The commercialization landscape, though growing, is hampered by the nuanced classification of products and the inherent challenges in protecting phytochemical intellectual property. Furthermore, manufacturing hurdles such as nanoparticle reproducibility, sterility, and regulatory-grade quality assurance create bottlenecks for scalability. At the systemic level, cost, education, and equitable access influence how these innovations are integrated into standard care. Together, these insights underscore that clinical translation of nano-phytomedicines must be navigated through a holistic lens one that merges bioengineering, regulatory science, public health policy, and patient-centric strategies to ensure global therapeutic impact.

## 8. Future Prospects and Emerging Trends

### 8.1. Smart and Responsive Nano-Phytomedicine Systems

Smart and responsive nano-phytomedicine systems represent a transformative frontier in targeted and controlled therapeutic delivery. These advanced nanocarriers are engineered to respond to specific endogenous or exogenous stimuli, allowing on-demand release of phytochemicals with improved spatiotemporal precision and reduced off-target effects [251]. Such stimuli-responsive systems can be designed to react to changes in pH, temperature, redox conditions, enzymatic activity, magnetic or electric fields, and light irradiation. In pathological environments, such as tumor tissues or sites of inflammation, microenvironmental triggers like acidic pH or elevated levels of reactive oxygen species (ROS) provide natural stimuli for smart nanocarrier activation [252]. For example, pH-sensitive polymeric nanoparticles encapsulating curcumin have demonstrated enhanced release specifically in acidic tumor microenvironments, minimizing systemic exposure and improving antitumor efficacy [253]. Similarly, redox-responsive systems exploit the higher intracellular glutathione concentration within cancer cells to trigger rapid phytochemical release from disulfide bond-containing nanocarriers [254]. Externally triggered systems offer the advantage of precise temporal control. Magnetic nanoparticles loaded with phytochemicals enable magnetically guided delivery and heat-triggered release (magneto-thermal therapy) [255] Light-responsive nanoformulations use near-infrared (NIR) irradiation to induce localized release, combining photothermal or photodynamic therapy with phytochemical activity [256]. Enzyme-responsive carriers leverage overexpressed enzymes such as matrix metalloproteinases (MMPs) in diseased tissues to initiate drug release, allowing selectivity and minimizing damage to healthy cells [257]. Integration of multiple stimuli-responsive elements within a single nanoplatform is gaining traction, enabling synergistic release mechanisms tailored to complex disease microenvironments [258]. This multi-responsive behavior enhances therapeutic efficacy and circumvents drug resistance mechanisms. Despite promising preclinical results, challenges remain for clinical translation, including ensuring biocompatibility of stimuli-sensitive materials, scalability of complex formulations, and reproducible stimuli responsiveness in heterogeneous human tissues [259]. Advanced characterization techniques and computational modeling aid in optimizing responsiveness and predicting in vivo behavior. In summary, smart and responsive nano-phytomedicine systems herald a new era of precision therapy by coupling the natural therapeutic benefits of phytochemicals with advanced stimuli-sensitive nanotechnology, offering improved efficacy, safety, and patient compliance [260].

### 8.2. Artificial Intelligence and Computational Modeling in Formulation Design

Artificial intelligence (AI) and computational modeling have emerged as indispensable tools accelerating the rational design and optimization of nano-phytomedicine formulations. By leveraging machine learning (ML), deep learning, and advanced simulation algorithms, researchers can predict nanocarrier properties, drug release kinetics, and biological interactions, reducing trial-and-error experimentation and expediting development timelines [261]. AI-driven models analyze vast datasets from formulation experiments, physicochemical characterizations, and biological assays to identify critical parameters influencing nanoparticle size, stability, drug loading, and release profiles [262]. For example, ML algorithms have been successfully applied to optimize polymeric nanoparticle formulations encapsulating quercetin by predicting optimal polymer ratios and processing conditions to maximize encapsulation efficiency and controlled release [263]. Computational fluid dynamics (CFD) simulations and molecular dynamics (MD) modeling provide atomistic insights into nanoparticle assembly, phytochemical-polymer interactions, and membrane penetration mechanisms [264]. Such simulations enable the rational selection of materials and design features to enhance bioavailability and targeting. AI also facilitates predictive toxicology by modeling potential adverse effects based on nanoparticle physicochemical properties, helping to screen formulations before in vivo testing [265]. In the clinical realm, AI models analyze patient data to predict individual responses to nano-phytomedicines, supporting personalized treatment strategies [266]. Integration of AI with high-throughput experimental platforms enables rapid iterative design cycles, drastically reducing development costs and timelines [267]. Furthermore, natural language processing (NLP) tools mine scientific literature and patent databases to identify novel phytochemicals and nanocarrier combinations with therapeutic potential [268]. Challenges include the need for high-quality, standardized datasets and the interpretability of AI models to ensure reliable decision-making [269]. Interdisciplinary collaboration among formulation scientists, data scientists, and clinicians is essential to realize the full potential of AI in nano-phytomedicine design. Overall, AI and computational modeling constitute a paradigm shift toward data-driven, efficient, and precision formulation of nano-phytomedicines, accelerating their translation from concept to clinical reality [270].

### 8.3. Personalized Nano-Phytotherapy and Precision Medicine Approaches

The concept of personalized medicine tailoring therapeutic interventions to individual genetic, phenotypic, and environmental profiles is gaining momentum in phytomedicine, with nanoformulations playing a crucial enabling role [271]. Personalized nano-phytotherapy seeks to optimize phytochemical dosage, delivery, and combinations based on patient-specific characteristics, maximizing efficacy while minimizing adverse effects. Variability in patient metabolism, gut microbiota composition, immune status, and disease heterogeneity influences phytochemical pharmacokinetics and pharmacodynamics [272]. Nanocarriers can be engineered to address these interindividual differences by modulating release kinetics, targeting capabilities, and combination therapies. Advanced diagnostic tools, including genomic sequencing, metabolomics, and biomarker profiling, facilitate patient stratification and identification of responders to specific nano-phytomedicines [273]. For example, patients with specific polymorphisms affecting curcumin metabolism may benefit from formulations with enhanced stability or targeted delivery to bypass metabolic degradation. Combining nano-phytomedicines with companion diagnostics enables real-time monitoring of therapeutic response and dynamic adjustment of dosing regimens [274]. Theranostic nanocarriers integrating imaging agents with phytochemicals allow simultaneous diagnosis and treatment, enhancing precision [275]. Emerging technologies such as 3D bioprinting and microfluidic organ-on-chip platforms enable preclinical testing of personalized nano-phytomedicines on patient-derived cells and tissues, improving prediction of clinical outcomes [276]. However, challenges include the complexity of integrating multi-omics data into treatment decisions, the need for robust clinical validation, and ethical considerations around patient data privacy [277]. Regulatory frameworks must evolve to accommodate personalized phytotherapy approaches and facilitate their clinical adoption. In conclusion, personalized nano-phytotherapy represents a promising convergence of nanotechnology, phytochemistry, and precision medicine, offering tailored interventions that optimize patient outcomes and heralding a new standard in natural product therapeutics [278].

### 8.4. Sustainable and Green Nanotechnology Integration

Sustainability and environmental considerations are increasingly critical in the development of nano-phytomedicines, prompting integration of green chemistry principles and eco-friendly nanotechnologies [279]. Sustainable nanotechnology aims to minimize environmental impact throughout the life cycle of nanocarriers—from raw material sourcing and synthesis to manufacturing, usage, and disposal [280]. Green synthesis methods employing plant extracts, bacteria, fungi, or algae as reducing and stabilizing agents have gained prominence for fabricating metallic and polymeric nanoparticles, avoiding toxic chemicals and high-energy processes [281]. Such biogenic nanoparticles often display enhanced biocompatibility and therapeutic efficacy due to their surface biomolecules and eco-friendly synthesis routes [282]. In addition, solvent-free or aqueous-based nanoparticle fabrication methods reduce hazardous waste and occupational risks [283]. Utilizing renewable and biodegradable polymers (e.g., chitosan, alginate, cellulose derivatives) as nanocarrier matrices aligns with circular economy principles and reduces persistence of nano-waste in the environment [284]. Life cycle assessment (LCA) tools are increasingly applied to evaluate the environmental footprint of nano-phytomedicine production, guiding sustainable design and manufacturing decisions [285]. Strategies such as energy-efficient synthesis, waste valorization, and green purification technologies contribute to reducing carbon footprint and resource consumption [286]. Moreover, regulatory agencies and funding bodies encourage development of sustainable nanomedicines, emphasizing responsible innovation and societal impact [287]. Public perception and acceptance also favor environmentally conscious products, enhancing market potential. However, regulatory acceptance of green-synthesized nanoformulations is still evolving, as the reproducibility, consistency, and scalability of biogenic synthesis remain key challenges. Safety concerns arise from batch-to-batch variability and the undefined nature of surface biomolecules, which complicate toxicological assessment and regulatory classification. Standardization of green synthesis protocols and characterization techniques is necessary to ensure compliance with Good Manufacturing Practice (GMP) guidelines and to satisfy the quality control demands of regulatory authorities. Additionally, nanocarriers developed through green routes must undergo rigorous preclinical testing to confirm their long-term safety and environmental impact, especially when biodegradable carriers are involved in systemic administration. Challenges include balancing sustainability with cost, scalability, and maintaining high-quality standards for therapeutic efficacy [288]. Furthermore, internationally harmonized regulatory frameworks specific to green nanomedicine are currently lacking, creating uncertainty in approval pathways. As a result, developers must proactively engage with regulatory bodies during early development stages to align their eco-friendly approaches with existing guidelines and anticipate future requirements. Continuous innovation, interdisciplinary collaboration, and policy support are essential to embed sustainability in nano-phytomedicine development fully. In summary, integrating sustainable and green nanotechnology principles into nano-phytomedicine research and production ensures that advances in natural product therapeutics align with global environmental and societal goals, fostering responsible innovation and long-term viability [289]. A parallel emphasis on regulatory preparedness and quality assurance will be pivotal to transforming green innovations into clinically approved, market-ready nano-phytomedicine products. Table 7 highlights how nano-phytomedicine is evolving beyond conventional delivery systems toward highly intelligent, patient-specific, and environmentally conscious platforms. The integration of external triggers and smart responses reflects a move toward greater therapeutic precision. Meanwhile, computational approaches are reshaping formulation design, offering data-driven efficiency. Personalized strategies underscore the shift toward individualized care, while eco-friendly fabrication methods respond to growing demands for sustainability in healthcare innovation. These trends collectively represent a multidimensional advancement in both scientific rigor and translational potential.

## 9. Conclusions

In recent years, the convergence of phytochemistry and nanotechnology has ushered in a new paradigm in natural product therapeutics. The encapsulation of plant-derived bioactive compounds within diverse nanocarrier systems has substantially enhanced their solubility, stability, bioavailability, and targeted delivery capabilities. Advances in smart and stimuli-responsive nanocarriers have enabled controlled, site-specific release, maximizing therapeutic efficacy while minimizing systemic toxicity. Additionally, integration of computational modeling and artificial intelligence has accelerated formulation optimization and personalized treatment approaches. Collectively, these innovations have elevated nano-phytomedicines from traditional herbal remedies to precision nanotherapeutics with vast potential across a broad spectrum of diseases. Despite significant progress, challenges remain that hinder the full clinical translation of nano-phytomedicines. Complexities in standardizing botanical extracts, achieving scalable and reproducible manufacturing, and ensuring long-term safety and regulatory compliance persist as key obstacles. Furthermore, limited clinical trial data and variability in patient responses call for more robust validation frameworks. However, these limitations present opportunities for interdisciplinary collaboration, innovative formulation strategies, and integration of emerging technologies such as machine learning and advanced diagnostic tools. Continued efforts to address these gaps will unlock the full therapeutic potential of nano-phytomedicines and enable their broader acceptance within mainstream healthcare. The future integration of nano-phytomedicines into global healthcare systems promises to redefine natural product-based therapy by combining traditional wisdom with cutting-edge nanotechnology and personalized medicine. By overcoming current barriers and aligning development with sustainability and accessibility goals, nano-phytomedicines can offer cost-effective, safe, and efficacious treatment alternatives, especially for chronic and complex diseases. Their successful adoption will depend on rigorous scientific validation, clear regulatory pathways, and education to foster acceptance among clinicians and patients. Ultimately, the synergy of phytochemistry and nanomedicine holds great promise for advancing holistic, patient-centered care worldwide.

## Figures and Tables

**Table 1:** Comparative overview of major phytochemical classes, therapeutic properties, and delivery challenges.

Phytochemical Class	Example Compounds	Primary Therapeutic Properties	Delivery Challenges	References
**Polyphenols**	Curcumin, Resveratrol, EGCG	Antioxidant, anti-inflammatory, anticancer	Poor aqueous solubility, rapid metabolism, low absorption	[30,32]
**Alkaloids**	Berberine, Morphine, Vincristine	Antibacterial, antidiabetic, anticancer	Narrow therapeutic index, high toxicity at higher doses	[28,37]
**Terpenoids**	Limonene, Paclitaxel, Carotenoids	Anticancer, antimicrobial, cardioprotective	Oxidative instability, poor bioavailability	[29]
**Flavonoids**	Quercetin, Kaempferol, Naringenin	Antioxidant, neuroprotective, anti-inflammatory	Low membrane permeability, rapid phase II metabolism	[30,37]
**Glycosides**	Digoxin, Sennosides, Stevioside	Cardiotonic, laxative, metabolic regulation	Limited stability in GI tract, slow release kinetics	[31]

EGCG = Epigallocatechin gallate, GI tract = Gastrointestinal tract.

**Table 2:** Comparative analysis of nanocarrier systems for phytochemical delivery.

Nanocarrier Type	Key Features	Advantages in Phytochemical Delivery	Limitations/Challenges	Representative Applications	References
**Liposomes**	Phospholipid bilayer vesicles with hydrophilic core and lipophilic shell	Biocompatibility, can carry both hydrophilic and lipophilic phytochemicals, enhanced absorption	Physical instability, oxidation of lipids, rapid clearance without PEGylation	Curcumin, resveratrol, EGCG for cancer and inflammation	[49]
**Solid Lipid Nanoparticles (SLNs)**	Nanocarriers made of solid lipids stabilized by surfactants	High loading of lipophilic phytochemicals, protection from degradation, scalable production	Limited drug loading, potential for polymorphic transitions affecting stability	Silymarin, quercetin for liver protection and metabolic disorders	[52,53,54]
**Nanostructured Lipid Carriers (NLCs)**	Mix of solid and liquid lipids to form a less ordered matrix	Improved drug loading, prolonged release, better stability than SLNs	Complex formulation optimization needed, possibility of lipid phase separation	Berberine, naringenin for metabolic and cardiovascular diseases	[55,56]
**Natural Polymeric NPs**	Polysaccharide (e.g., chitosan, alginate) or protein-based carriers	Biodegradable, mucoadhesive, enhances intestinal uptake and stability	Batch variability, lower mechanical strength than synthetic carriers	Chitosan-curcumin NPs for oral anti-inflammatory therapy	[63,64]
**Synthetic Polymeric NPs**	PLGA, PCL, PEG-based systems designed via emulsion or nanoprecipitation	Customizable release profile, high encapsulation efficiency, FDA-accepted polymers	Requires organic solvents, potential accumulation of degradation products	PLGA-resveratrol or quercetin NPs for cancer and neurodegenerative diseases	[67,68]
**Inorganic NPs—Metal**	Gold (AuNPs), Silver (AgNPs), synthesized via green or chemical methods	Excellent stability, imaging compatibility, tunable size/shape, antimicrobial synergy with phytochemicals	Potential cytotoxicity, accumulation risk, surface functionalization required for targeting	AgNP–berberine conjugates for antimicrobial activity	[76,77]
**Inorganic NPs—Metal Oxide**	ZnO, Fe_3_O_4_, TiO_2_, often used with plant extract functionalization	Intrinsic bioactivity (e.g., ROS generation), magnetic guidance, enhanced dissolution	Long-term toxicity concerns, environmental persistence	ZnO–curcumin for wound healing, Fe_3_O_4_–EGCG for brain targeting	[81]
**Inorganic NPs—Silica (MSNs)**	Mesoporous silica with tunable pore size and surface area	High surface loading, pH-sensitive release, good biocompatibility	Non-biodegradable, risk of particle accumulation and chronic exposure	MSNs with polyphenols for anticancer or gut delivery	[83]
**Hybrid Nanocarriers**	Lipid-polymer combinations, metal-lipid or polymer-metal hybrids	Combines benefits of each system, controlled release, structural stability, multi-functional capability	Complex synthesis, scalability challenges	PLGA–lipid hybrid loaded with curcumin for dual-release in tumors	[87]
**Stimuli-Responsive Systems**	pH, redox, enzyme, temperature, or light-triggered release platforms	On-demand release at disease sites, reduced systemic toxicity	Stimuli-specific tuning required, clinical validation is limited	pH-sensitive quercetin NPs for tumor microenvironments	[88,89]
**Surface-Functionalized NPs**	Nanoparticles modified with ligands (folate, peptides, antibodies, aptamers) for active targeting	Receptor-specific delivery, improved uptake in diseased cells/tissues	Ligand synthesis cost, potential immune response, ligand detachment in vivo	Folic acid-decorated NLCs for ovarian cancer therapy	[90,91]

**Table 3:** Comparative insights into the molecular mechanisms of action of nano-phytomedicine.

Mechanism	Key Features	Nano-Phytomedicine Role	Advantages of Nanoformulation	Representative Phytochemicals	References
Cellular Uptake and Intracellular Trafficking	Involves endocytosis (clathrin, caveolin, macropinocytosis), endosomal escape, and organelle targeting	Enables cytosolic/nuclear delivery; bypasses degradation; improves uptake in target cells	Enhanced retention, subcellular targeting, P-gp bypass, prolonged release	Curcumin, resveratrol, quercetin	[101,102]
Redox Balance and Antioxidant Pathways	Modulation of oxidative stress via Nrf2 pathway, ROS scavenging, and GSH regulation	Improves intracellular antioxidant levels, activates protective genes (e.g., HO-1, NQO1)	Site-specific ROS scavenging, redox-responsive release, mitochondrial targeting	EGCG, curcumin, sulforaphane	[106,107,108,109,110]
Apoptosis, Proliferation and Inflammation	Targets intrinsic/extrinsic apoptotic pathways, NF-κB, and cytokine networks	Triggers caspase cascade, downregulates Bcl-2, inhibits COX-2 and TNF-α production	Selective cancer cell apoptosis, reduced systemic toxicity, enhanced anti-inflammatory effects	Berberine, apigenin, quercetin	[111]
Signaling Pathway Regulation	Interference with PI3K/Akt, MAPK, and NF-κB cascades	Inhibits survival/proliferation signals; enhances apoptosis and immune responses	Sustained pathway inhibition, synergistic effects with ligands or co-drugs	Kaempferol, luteolin, curcumin	[118],
Epigenetic and Genomic Regulation	Involves DNA methylation, histone acetylation, miRNA modulation	Modulates DNMTs, HDACs, and epigenetic markers for long-term gene expression control	Nuclear delivery, miRNA-targeted therapy, chromatin remodeling	Resveratrol, sulforaphane, EGCG	[124]

P-gp: P-glycoprotein; ROS: Reactive oxygen species; Nrf2: Nuclear factor erythroid 2-related factor 2; DNMTs: DNA methyltransferases; HDACs: Histone deacetylases; GSH: Glutathione; HO-1: Heme oxygenase-1; NQO1: NAD(P)H quinone dehydrogenase 1.

**Table 4:** Comparative overview of nano-phytomedicine applications in human health.

Therapeutic Area	Mechanism of Action/Benefit	Nanocarrier Strategies	Representative Phytochemicals	References
**Cancer Therapy**	Tumor targeting via EPR effect, apoptosis induction, ferroptosis, and anti-angiogenesis	Liposomes, polymeric nanoparticles, gold nanoshells; HER2, folate-targeted systems	Curcumin, resveratrol, EGCG, paclitaxel precursors	[130,131,132,133,134,135,136]
**Cardiovascular Health**	Anti-atherogenic (↓ LDL oxidation, ↓ VSMC proliferation), antihypertensive (↑ NO release, ↓ vascular resistance), anti-inflammatory	Nanoemulsions, lipid-polymer hybrids, magnetic targeting nanoparticles	Resveratrol, catechins, naringenin	[137,138,139,140,141,142]
**Neurodegenerative Disorders**	BBB penetration, microglial modulation, anti-amyloid aggregation, antioxidant and anti-inflammatory neuroprotection	Liposomes, polymeric nanoparticles with surfactant/ligand coating (e.g., polysorbate-80, transferrin)	Curcumin, resveratrol, EGCG	[143,144,145,146]
**Metabolic Disorders**	Glycemic control, insulin sensitization, adipogenesis inhibition, lipid modulation, anti-inflammatory and thermogenic effects	Chitosan, silver NPs, lipid-polymer hybrids, nanoencapsulation for GI protection and sustained release	Berberine, quercetin, curcumin, silymarin, catechins	[147,148,149,150,151,152]
**Infectious Diseases**	Antibacterial (biofilm disruption, membrane damage), antiviral (entry blockade, protease inhibition), antifungal (membrane disruption), resistance mitigation (efflux inhibition)	Metal–phytochemical NPs (e.g., Ag, ZnO), polymeric nanocapsules, gold conjugates, inhalable/topical delivery formats	Curcumin, baicalin, quercetin, thymol, eugenol	[153,154,155,156]

**Table 5:** Advanced pharmacokinetic and toxicological determinants of nano-phytomedicine performance.

Pharmacological/Toxicological Dimension	Scientific Insight and Mechanistic Role	Nano-Phytomedicine Strategy/Advantage	Emerging Barriers/Optimization Needs	References
**Kinetic Modulation via Nano-Architecture**	Nanoform morphology (e.g., core-shell, hollow, matrix) influences degradation and release rates through interfacial drug diffusion and structural disintegration	PLGA matrix vs. lipid vesicles: predictable vs. burst release kinetics; core–shell hybrids improve tunability	Nano–drug mismatch (e.g., rapid-release from hydrophobic matrices), cross-linking irregularity	[156,159,160]
**Gastrointestinal Stability and Transit Behavior**	Nano-encapsulation delays premature phytochemical degradation by gastric enzymes and bile salts, improving residence time and epithelial transport	Enteric-coated or mucin-adhering nanoparticles preserve payload integrity and enhance absorption	GI transit variability, impact of digestive enzymes on carrier integrity	[159,163,165]
**Epithelial Transport and Endocytosis Pathways**	Nanoform size/charge influences endocytic route: clathrin/caveolae-mediated or macropinocytosis; smaller particles often cross via paracellular or tight junctions	Surface-tuned nanoparticles exploit paracellular pathways (e.g., chitosan opens tight junctions), enhancing transepithelial flux	Excessive opening of junctions may increase toxicity; variability in transporter expression among individuals	[163,165,166]
**Organ-Specific Accumulation Dynamics**	Organotropism is governed by anatomical microenvironment, vascular permeability, and nanoparticle deformability or elasticity	Flexible or elongated nanoparticles exhibit greater tumor margination and accumulation; stealth PEG coating extends circulation	Shape-induced immune clearance, variability in microvascular permeability in disease states	[171,173,181]
**Mononuclear Phagocyte System (MPS) Interactions**	Recognition by Kupffer cells and splenic macrophages remains a major clearance mechanism, influenced by opsonization and protein corona formation	PEGylation or biomimetic cloaking (e.g., RBC membrane) reduces MPS uptake and enhances plasma half-life	Accelerated clearance after repeated dosing (anti-PEG antibodies); need for personalized stealth coatings	[174,176,186]
**Cytokine and Complement Response Mechanisms**	Nanoparticles can activate TLRs or complement system, leading to cytokine storms or hypersensitivity depending on their physicochemical profile	Using neutral/zwitterionic polymers lowers immune activation; shape and surface control mitigate pro-inflammatory signaling	Risk of chronic low-grade inflammation; inter-individual differences in immune recognition pathways	[191,193,198]
**Metabolic Fate and Excretion Pathways**	Surface functionalization and hydrodynamic diameter define renal (≤10 nm) vs. hepatobiliary clearance (≥50 nm); metabolism by macrophage enzymes modifies excretion patterns	Size-tuned nanoparticles designed for controlled hepatic metabolism or renal filtration; active efflux modulation is possible	Risk of hepatic overloading or kidney retention with chronic use; lack of data on long-term excretion metabolites	[169,184,199]
**Standardization for Regulatory Approval**	ISO 10993 and ICH M3(R2) guidelines emphasize safety, biodegradation, genotoxicity, and immunotoxicity profiling in nanomedicine development	Nanoformulations undergo systematic hemolysis, cytotoxicity, and cytokine profiling prior to preclinical testing	Absence of phytochemical-specific nanotoxicity thresholds; inter-lab variability in compliance methodologies	[28]

PLGA: Poly(lactic-co-glycolic acid); PEG: Polyethylene glycol; MPS: Mononuclear Phagocyte System; RBC: Red Blood Cell; ISO: International Organization for Standardization; TLR: Toll-like receptor.

**Table 6:** Key clinical and translational insights in nano-phytomedicine development.

Focus Area	Scientific Highlights	Challenges and Strategic Needs	References
Preclinical and Clinical Trials	Demonstrated enhanced efficacy in murine models (e.g., liposomal curcumin, polymeric resveratrol); improved ADME profiles via nanoencapsulation	Limited human trials, complex regulatory pathways, need for predictive preclinical models and adaptive clinical designs	[218,219,220,221,222,223,224,225,226]
Commercial Products and Patent Landscape	Several liposomal/polymeric products on market as supplements; rising patents in targeted nanocarriers and controlled release technologies	Regulatory heterogeneity between supplements and drugs; IP protection of complex phytochemical systems remains difficult	[227,228,229,230,231,232,233]
Scale-Up and Manufacturing Barriers	Techniques like high-pressure homogenization and microfluidics can enhance reproducibility; PAT tools support QbD compliance	Batch-to-batch variation, costly GMP production, lack of industrial scalability for many prototype nanoformulations	[234,235,236,237,238,239,240,241,242,243]
Cost, Accessibility, and Healthcare Acceptance	Nanoformulations may reduce dosing and improve outcomes; potential for long-term cost-effectiveness with fewer side effects and improved patient adherence	High initial cost, unequal access in low-income regions, lack of awareness and acceptance among healthcare providers and patients	[244,245,246,247,248,249,250]

ADME: Absorption, Distribution, Metabolism, and Excretion; QbD: Quality by Design; PAT: Process Analytical Technology; GMP: Good Manufacturing Practice; IP: Intellectual Property.

**Table 7:** Emerging trends and translational strategies in nano-phytomedicine.

Emerging Trend	Key Features	Translational Advantages	References
Smart and Responsive Nano-Phytomedicine Systems	Stimuli-responsive release triggered by pH, ROS, enzymes, light, or magnetic fields; enables site-specific and on-demand phytochemical delivery.	Enhanced spatiotemporal control, reduced off-target toxicity, and improved efficacy in complex disease microenvironments.	[251,252,253,254,255,256,257,258,259,260]
Artificial Intelligence and Computational Modeling in Formulation Design	Machine learning and modeling tools used to predict formulation outcomes, optimize nanocarrier parameters, and reduce trial-and-error cycles.	Accelerated development timelines, reduced costs, improved reproducibility, and predictive safety/toxicity screening.	[261,262,263,264,265,266,267,268,269,270]
Personalized Nano-Phytotherapy and Precision Medicine Approaches	Stratified treatments using biomarker profiling, genomics, and pharmacogenomics; theranostics and companion diagnostics enhance personalization.	Customized therapies for heterogeneous patient populations, improved adherence, reduced side effects, and dynamic dose optimization.	[271,272,273,274,275,276,277,278]
Sustainable and Green Nanotechnology Integration	Green synthesis using biogenic routes; biodegradable carriers; aqueous-based processes and LCA-driven design minimize environmental impact.	Improved public acceptance, regulatory alignment, reduced ecological burden, and compliance with sustainability mandates.	[279,280,281,282,283,284,285,286,287,288,289]
